# Open and shared sustainable mega-constellation

**DOI:** 10.1093/nsr/nwaf344

**Published:** 2025-08-23

**Authors:** Jun Yang, Junxiang Qin, Xiye Guo, Zhixi Yang, Ganhua Ye, Xuan Li, Xiaotian Ma, Suyang Liu, Sili Liu, Xianbin Li, Zhijun Meng, Chao Zhou, Zhi Qu, Mei Hu, Jianyun Chen

**Affiliations:** College of Intelligence Science and Technology, National University of Defense Technology, China; Sixty-third Research Institute, National University of Defense Technology, China; College of Intelligence Science and Technology, National University of Defense Technology, China; Sixty-third Research Institute, National University of Defense Technology, China; Sixty-third Research Institute, National University of Defense Technology, China; College of Intelligence Science and Technology, National University of Defense Technology, China; College of Intelligence Science and Technology, National University of Defense Technology, China; College of Intelligence Science and Technology, National University of Defense Technology, China; Sixty-third Research Institute, National University of Defense Technology, China; College of Intelligence Science and Technology, National University of Defense Technology, China; College of Intelligence Science and Technology, National University of Defense Technology, China; College of Intelligence Science and Technology, National University of Defense Technology, China; College of Intelligence Science and Technology, National University of Defense Technology, China; College of Intelligence Science and Technology, National University of Defense Technology, China; College of Intelligence Science and Technology, National University of Defense Technology, China

## Abstract

The paper proposes an open and shared sustainable mega-constellation with the "cloud-pool-terminal" paradigm and "Sensors+Network+AI" architecture, reshaping the satellite service mode and effectively solving the challenges of space environment sustainability.

The Earth’s orbital space is becoming increasingly crowded due to the accelerated deployment of mega-constellations such as Starlink [1]. From 2017 to 2024, more than 300 constellation filings were submitted to the International Telecommunication Union, with a total of over one million satellites [2]. It is predicted that by 2030, there will likely be more than 100 000 satellites in low Earth orbit [3]. However, low-orbit space is not renewable, and its capacity is extremely limited [2]. Considering the safety distance required for satellite orbital motion, with a minimum of 50 km between satellites both within and between orbital layers, low-orbit space from 300 to 2000 km can only accommodate 175 000 satellites [4]. Therefore, the demand for orbit space has far exceeded the capacity.

The proliferation of satellite constellations can exacerbate the problem of space sustainability. Although it can expand service accessibility, it brings unsustainable risks, including orbital congestion, radio-frequency interference, collision risks and geopolitical competition [5], while generating environmental externalities such as atmospheric pollution, compromised astronomical observations and crewed mission hazards. The United Nations Outer Space Treaty stipulates that orbital resources are assets shared globally and as a scientific, cultural and operational heritage [6]. However, due to the closure of satellite resources, the isolation of the constellation architecture and the solidification of the service model, it

is difficult for existing constellation systems to interconnect and share resources. Each country has to build its own space systems, and this large-scale deployment of satellites naturally leads to the problem of space sustainability.

The root cause of the space unsustainability problem of mega-constellations is the fixed service model and low resource utilization. The space systems’ resource utilization, driven by satellite system isolation and inefficient wide-area service provision, is less than 10% [7]. As we all know, the concept of interconnection and open sharing of international internet standards has revolutionized the utilization of computing resources and changed the service model. To address the issue of space unsustainability, drawing inspiration from the internet’s principle of a single global network, we propose an open and unified intelligent satellite (iSat) architecture along with an intelligent space system (ISS) based on the ‘cloud-pool-terminal’ (CPT) paradigm; see Fig. 1b, c and d, respectively. We extend the theory of multi-layer and multi-sensor orbit collaborative coverage, and use the two architectures to drive the transformation of space systems from country-specific large-scale deployments to a new, open and shared paradigm. The paradigm supports ISS to be a service-oriented, mission-centered space system. Furthermore, the constellations not only serve ground terminals, but also provide connectivity to satellites in lower orbits or high orbits [8]. This connection would greatly improve

satellite resource-sharing effectiveness throughout space systems. For example, ISS can provide full-time space-based connectivity services for China’s space station. This paradigm can also meet global service demands while keeping the number of satellites stable, thereby addressing the problem of sustainable development in the space environment and fostering a global community with a shared future for humanity. Thus, we call it an open and shared sustainable mega-constellation (OSSMC).

**Figure 1.**
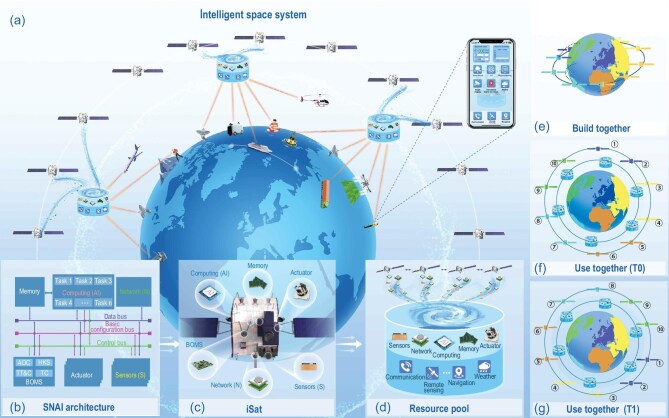
The co-construction, co-sharing and co-management paradigm based on the intelligent space system and intelligent satellites (a). Based on an open and unified SNAI architecture (b), iSat shares resources and builds a shared space resource pool (c, d), allowing countries around the world to jointly build a space system (e), join the space network to share satellite resources (f) and use the resource pool as needed (g). In (f) and (g), the different colors represent users in different areas, and the circled numbers denote the identification numbers of satellites. (f) and (g) represent the same satellites at different times (T0 and T1). Different types of users can access satellite networks at any point and anytime around the world, without being constrained by orbit, and enjoy real-time satellite resource services worldwide.

As shown in Fig. 1, the OSSMC is enabled by the iSat and ISS, both operating within the CPT paradigm. This paradigm establishes a globally shared satellite resource pool, enabling on-demand access to space assets regardless of orbital configuration, while promoting collaborative multi-national construction and equitable participation in system governance—akin to cloud computing principles—thereby replacing redundant national constellations with a unified infrastructure that prevents space privatization and ensures sustainable, peace-oriented resource utilization.

The improvement in the resource efficiency of the ISS comes from its ability to be a task-oriented, on-demand service that delivers an immediate response. To achieve this, the ISS must shift resource requests and services from being passive to active [9], as illustrated by the three user service scenarios for different regions in Fig. 1a. Therefore, we simplify the control, computing, communication, remote sensing and navigation functions of satellites into three core elements—sensors, networks and artificial intelligence (AI)—and design a unified satellite architecture based on ‘Sensors+Network+AI’ (SNAI). Openness is reflected in the fact that the resources inside and between satellites can be dynamically shared as needed, which provides on-demand resources for the tasks randomly distributed by the space system. This greatly reduces the idle time of resources, improving the utilization rate of satellite resources, and serving more users.

The ISS architecture incorporates a space cloud platform on resource-rich satellites to support demand-driven resource allocation, intelligent scheduling and global ‘one-point access’ through a unified resource pool, enabled by iSat’s task-level or signal-level cooperation and customizable time-synchronized services. This software-defined paradigm dynamically assigns satellites to regions based on user needs, eliminating redundant national constellations while ensuring continuous coverage as satellites transition between service areas. This is exactly the usage scenario shown in Fig. 1e, f and g.

We evaluated sustainability gain, service ability, system cost and task efficiency of the proposed ISS based on the CPT architecture with other space systems to fully demonstrate the advantages of this development paradigm. See the online supplementary material for a detailed experimental design and analysis. We assessed and analyzed the sustainability of the ISS and existing typical space systems. We found the orbit impact score (OIS) [10] for four space systems and three scenarios. It can be observed that the ISS reduces the space volume collision rate by 28.7% compared with the combined system, equivalent to a reduction of more than one-quarter. The OIS of the ISS decreased by 53.15%, equivalent to a reduction of more than half.

To meet the demand for equal coverage and average communication capacity greater than 100 Mbps, the ISS requires only 1/58 of the satellites needed by Starlink. In densely populated areas, communication services can be provided by ground communication infrastructure, with satellite communication serving as a supplement to ground communication and serving remote areas. System performance and cost evaluation show that ISSs can reduce the cost by 19.15%, but still improve the constellation coverage and global geometric dilution of precision (GDOP), compared with independent functional domains and combined space systems. The improvement in GDOP by the ISS is significantly better than that of Centispace systems, with an average improvement of 51.07%, which is 19.05% higher than that of Centispace systems. Hawkeye 360 only covers key areas on level 2 or above, while the ISS can have global coverage with a minimum weight of 15.

In terms of task effectiveness, we theoretically proved and verified the sustainable solution for the space environment using semi-physical simulations. Compared to traditional functional space systems, the ISS offers task-oriented and on-demand services. The CPT paradigm boosts the task success rate from 26.21% to 45.73% and reduces the failure rate from 51.45% to 1.64%. Although it brings issues like increased delay due to resource sharing, the overall improvement in resource utilization and service efficiency is notable. Analyses of energy and network utilization rates also show that the CPT paradigm is cost effective for satellites, given their solar-powered charging capabilities. The task success rate can be improved to 97% for randomly distributed tasks, and the task response time can be effectively improved.

This paper proposes OSSMC based on an SNAI satellite architecture and an ISS architecture, which provides solutions for the sustainability of the space environment. An interesting conclusion is that the global population of eight billion would require only about 50 000 satellites, rather than millions. The open and shared paradigm, supported by the theory of multi-layer and multi-sensor orbit collaborative coverage, is expected to transform large-scale deployment of low-Earth-orbit satellites into a space infrastructure that can meet diverse and personalized user needs worldwide, while avoiding the problem of the space environment becoming unusable after the deployment of one million satellites.

The CPT paradigm offers a solution that ensures the sustainable development of the space environment while maintaining the service level of space systems, enabling the realization of a sustainable mega-constellation with a shared future for humanity. The SNAI satellite architecture we propose may also change satellites like the von Neumann architecture; the CPT paradigm, like the internet and cloud computing, will change the industry and services of space systems.

## Supplementary Material

nwaf344_Supplemental_File
