# Supplementary material for: Open and shared sustainable mega-constellation
Source: Natl Sci Rev. 2025 Aug 23;12(11):nwaf344. doi: 10.1093/nsr/nwaf344 (PMC12527350; doi:10.1093/nsr/nwaf344)
Supplement: nwaf344_Supplemental_File [file nwaf344_supplemental_file.pdf]

Supplementary Materials for

Open and Shared Sustainable Mega-Constellation

Jun Yang[1]*†, Junxiang Qin[2]†*, Xiye Guo[1]*, Zhixi Yang[2], Ganhua Ye[2], Xuan Li[1], Xiaotian Ma[1], Suyang Liu[1], Sili Liu[2], Xianbin Li[1], Zhijun Meng[1], Chao Zhou[1], Zhi Qu[1], Mei Hu[1], Jianyun Chen[1]

[1] College of Intelligence Science and Technology, National University of Defense Technology, Changsha, China.
[2] The 63rd Research Institute, National University of Defense Technology, Nanjing, China.

Corresponding author: jyang@nudt.edu.cn; tanjunxiang12@nudt.edu.cn; nudtgxy@nudt.edu.cn

Materials and Methods

The paper innovates the theory of multiple resource coverage based on the concept of resource sharing by resetting the hardware architecture, space-based system architecture, and resource service mode of satellites, achieving the maximization of space-based resource utilization.

The supplementary materials consist of seven sections, which can be considered as three main parts: system design, experimental platform, and result analysis. The first and second sections are about system design, providing detailed introductions to satellite architecture and intelligent space system (ISS), especially constellation design. The third and fourth parts mainly introduce the experimental platform and some analysis and calculation methods adopted. Sections five to seven analyzed the experimental results from three aspects: sustainable gain of the system, system performance, and task effectiveness.

1. Satellite Design

ISat leverages the innovative SNAI (Sensors+Network+AI) architecture, integrating sensors for data acquisition, network for inter-satellite and ground connections, and AI-driven computing units for interface, control, and data processing. This design enhances resource sharing and task execution efficiency. The architecture of iSat essentially improves the scalability of satellite hardware and the flexibility of system resources[1].

The SNAI architecture, as shown in Fig. S1, encompasses sensors, network, computing unit, storage unit, basic maintenance unit, actuator and a set of communication buses. The 'S' in the SNAI architecture, which represents the sensor, serves as the initial point of satellite data acquisition and collection. It is responsible for acquiring and processing its own status data, or it may undertake the front-end signal processing and data acquisition of special services[1]. The 'N' refers to the network, which is a network module for the interconnection of nodes by satellite. It is used for measurement and communication, as well as for establishing inter-satellite and satellite ground network connections[5]. It serves as the fundamental element in the interaction of information and the maintenance of a benchmark in space and time. As a kind of satellite resource, the network is also managed by the computing unit to enable the on-demand scheduling of network resources. The 'AI' is embodied in the computing unit, which is a collection of general and professional processors on the satellite. Its function is to complete the calculation and processing of satellite interface, instruction, control, data, network and artificial intelligence engine. The basic on orbit maintenance system (BOMS) is the fundamental maintenance system for satellite's on-orbit operation. It encompasses a range of functions, including attitude and orbit control, measurement and control, and thermal control, with the objective of maintaining the satellite in a state of optimal on-orbit performance, ensuring uninterrupted communication and preventing any loss of functionality. It is imperative that the BOMS be designed with the utmost reliability and safety in mind, and the functions of this part are encrypted, reinforced and protected to avoid being changed by ordinary users at will to prevent single-event hardware failure and the potential loss or even permanent damage to the satellite. This module can not be changed by ordinary users when the satellite is leased. BOMS can only be accessed by the system administrator with the highest authority, while related software on other processors can configure access permissions based on user permissions to ensure satellite security.

The core of the open architecture of SNAI lies in its openness, as shown in Fig. S2. The traditional satellite architecture, is a kind of function satellite, whose resources are closed and cannot be shared. The openness of SNAI is reflected in that the satellite is divided into computing, network, storage, sensor, actuator and other units. Each unit interacts with each other through the bus. The unit is divided based on the logical functionality, which only ensures the module independence, but also enhances the robustness, and reconfigurability of the satellite. The resources inside and between satellites can be dynamically shared as needed, which provides on-demand resources for the tasks randomly distributed by the space system. This architecture is different from the traditional satellite with independent functional modules, allowing the satellite to provide in orbit redefined services in different regions and for different users, thus greatly reducing the idle time of resources, improving the utilization rate of satellite resources, and serving more users. As shown in Fig. S3c and Fig. S3f, intelligent satellite (iSat) in the ISS greatly improve the service time and quality of users by providing resource pool services for users.

The open architecture of SNAI has well solved the problems of compatibility, interoperability and sharing of different satellite hardware resources. The volume power consumption of satellites with different functions is different, and the configuration of resources such as sensors, calculation and storage is different. However, for the SNAI architecture, due to the standardized and modular hardware modules, as long as they meet the unified interface protocol standards, they can be integrated into the system to realize resource sharing and provide users with on-demand resource services.

The task migration of satellites can be divided into three stages. The first stage is the planning stage of satellite resource allocation. This stage obtains satellite task requirements and uses methods such as game theory[3] to plan resource allocation based on satellite resource status and satellite position. In the second stage, based on the planning results of task resource allocation, the management nodes of the satellite resource pool begin to store the status of each satellite and complete the transmission of tasks in the form of containers. In the third stage, tasks newly transmitted to the satellite begin to obtain the allocated resources and start corresponding task execution.

2. Constellation Design

The constellation design optimizes coverage by integrating multi-layer constellations, balancing cost and performance through cross-domain fusion. User demands are met via hierarchical collaborative reverse optimization, minimizing system costs while maximizing service performance. A novel multidisciplinary optimization method is introduced for constellation design, facilitating fast convergence and effective resource allocation. Sensor deployment optimization eliminates redundant coverage, enhancing service quality and reducing satellite numbers. This approach not only ensures optimal spatial-temporal resource utilization but also significantly reduces space debris, offering a sustainable solution for future satellite systems. The theoretical innovation lies in dynamic resource management and collaborative task execution, providing a robust framework for intelligent space systems.

The design process of the ISS is shown in Fig. S4. Firstly, configure the basic task requirements, such as whether the coverage target is regional or global, service requirements, such as navigation services for the global geometric dilution of precision (GDOP)[4], communication services for communication capacity and time, remote sensing services for

coverage and revisit time. Although the functions of the ISS can be defined by software in orbit, they are constrained by the sensors and processor carried by the satellite itself. So corresponding sensor constraints need to be provided. The next step is to set the basic parameter constraints of the constellation, such as orbit altitude, orbit inclination, etc. In terms of altitude, certain strategies can be set, such as setting the altitude range of high, medium, and low orbits and the proportion of each level. The basic parameters of constellations cannot be determined with specific values in the early stage, and an estimated range can be given. The more precise the range limitation, the greater the reduction in computational complexity. Based on the above input, we conducted a multidisciplinary optimization of constellation design and obtained a cross-domain-fusion-based constellation. Then in the reverse optimization stage, the deployment of satellites and sensors is first optimized based on resource coverage, and the satellites that can be merged are merged to reduce system costs. Finally, output the design results, which mainly include the basic parameter information of cross domain fusion constellations and the deployment information of sensors on each satellite.

The demand for user services is divided based on the number of people in the world[6]. According to the number of population[6], the world will be gridded. Densely populated areas have a high demand for space resources, necessitating high communication capacity and service quality[7]. However, the population has a high demand for space-based systems, including communication, navigation, remote sensing, and so forth[8]. Therefore, it is essential to design a multifunctional hybrid constellation. Subsequently, a constellation is designed for each area of coverage, and finally, several constellations are summarised and optimised. The multi-function hybrid universal constellation is a kind of cross-domain integration constellation. Through the cross domain integration of multi-layer constellations with different heights and functions, the constellation system can effectively play the advantages of large high orbit coverage area and high detection resolution through cooperative work[9]. Furthermore, due to the cooperation between satellites, the cross-domain fusion constellation can effectively reduce the system cost on the premise of meeting the performance requirements. This is achieved by effectively optimizing the number of satellites and the deployment of sensors. The cross-domain fusion constellation is of great significance for reducing the number of satellites in orbit and the number of debris in the space environment.

2.1 User Requirements

The Fig. S5 illustrates the current state of global human communication interconnection[10]. Consequently, in regions where Internet access is unavailable, the provision of satellite Internet services by space systems becomes a necessity. So, firstly, normalize the global world according to the population, and the demand map is:

$$DM\,(grid) = \sum_{grid=1}^{N_g} D(\mathrm{grid})R \tag{1}$$

In Eq. 1, N_g is the number of grid divisions, $D(grid)$ is the population in grid, and R is the service demand for space resources in the region. Furthermore, use D to represent the demand data volume, and B is the busy hour ratio, α is the safety factor that ensures the minimum service quality during peak periods, and η is the utilization rate. The demand of a group of users is the product of the average throughput of users and the total number of users[11].

$$R = \frac{D}{30}\cdot\frac{B}{24}\cdot\frac{1}{60\cdot 60}\cdot\frac{(1+\alpha)}{\eta} \tag{2}$$

The structure of the hierarchical collaborative reverse optimization method used in the design is shown in Fig. S6. In the system-level design, the optimization objective of general parameters is to minimize the system cost. The objective of the dedicated parameters is to achieve the target service performance of each subsystem functional domain under the constraint of meeting the system-level objectives, such as the largest communication capacity and the highest navigation accuracy. In hierarchical collaborative design, what is passed down at the system level is the expected value of the design vector $X_i(i=1,2,3,\cdots)$ [12], and what is passed up at the subsystem level is to make the actual value of the design vector X_i' as small as possible. In order to accelerate the convergence of the problem, the parameters related to the design vector trajectory can be transferred at the subsystem level to reduce the repeated search between subsystems[13].

In the system level design, the constellations of the three functional domains are integrated through the cost model to form a combined constellation. In the process of synthesis, the cost of a space system is primarily determined by two factors: the number of satellites and the cost of a single satellite. The cost of a single satellite is mainly constituted by the expenses incurred for the basic satellite components and sensors[14]. The established optimization model is as follows:

(1) The general parameter optimization is outlined below:

$$\min C_{\text{cost}}(X) \tag{3}$$

$$\min \sum_{j=1}^{n}(X_j - X'_{1,j})^2 + \sum_{j=1}^{n}(X_j - X'_{2,j})^2 + \sum_{j=1}^{n}(X_j - X'_{3,j})^2 + \sum_{j=1}^{n}(X_j - X'_{4,j})^2 \tag{4}$$

(2) The communication domain design is as follows:

$$\min \sum_{j=1}^{n}(X_j - X'_{1,j})^2 \tag{5}$$

$$\text{s.t.} \quad k_{\min}^{com} \geq 1 \tag{6}$$

$$Cov^{\text{com}} \geq Cov_{\text{request}}^{\text{com}} \tag{7}$$

$$\alpha \geq 5° \tag{8}$$

The aforementioned constraint indicates that the minimum coverage $k_{\min}^{com}$ of the communication domain constellation is greater than or equal to one[15], and the coverage area Cov^{com} must exceed $Cov_{\text{request}}^{\text{com}}$. The basic constraint generally takes a value of 95%, and the minimum elevation angle of the user α can not be less than 5° [(16)].

(3) The navigation domain design is as follows:

$$\min \sum_{j=1}^{n}(X_j - X'_{2,j})^2 \tag{9}$$

$$\text{s.t.} \quad \text{GDOP} < \text{GDOP}_{\text{request}} \tag{10}$$

$$k_{\min}^{\text{nav}} \geq 4 \tag{11}$$

The above constraints GDOP indicate that the navigation domain constellation must meet the constraints $\text{GDOP}_{\text{request}}$, $k_{\min}^{\text{nav}}$ generally requiring a minimum coverage of less than five and a minimum coverage of four[17].

(4) The remote sensing domain design is as follows:

$$\min \sum_{j=1}^{n}(X_j - X'_{3,j})^2 \tag{12}$$

$$\text{s.t.} \quad Cov^{\text{rem}} > Cov^{\text{rem}}_{\text{request}} \tag{13}$$

$$\theta_{\min} < \theta < \theta_{\max} \tag{14}$$

$$E_{rsl} \le E_{\text{request}} \tag{15}$$

The constraint in Eq. 15 above indicates that the field of view angle of the remote sensing sensor is constrained within a certain range $[\theta_{\min}, \theta_{\max}]$, while meeting the designed coverage $Cov^{\text{rem}}_{\text{request}}$ and resolution constraints E_{request} [18].

If there are other functional domain designs, they would also be represented using $\min \sum_{j=1}^{n}(X_j - X'_{4,j})^2$ and $g(x) \le 0$ to participate in constellation design[19]. The expansion of other functional domains can be designed according to the requisite specifications, as this method does not limit the number of functional domains involved in collaborative optimization. The paper only focuses on design optimization for the most common communication, navigation, and remote sensing.

2.2 Subsystem Level Discipline Design Method

The basic constellation is the constellation generated in the first stage, which contains satellite constellations in different disciplines. This paper divides the design of intelligent space systems (ISS) into two levels: system level and subsystem level. In the process of completing the constellation design of their respective functional areas, subsystem-level disciplines share variables with other subsystem-level disciplines and system-level disciplines to achieve the goal of collaborative optimization.

(1) Method of Generating Communication Constellation

In satellite ground communication, users are primarily concerned with the communication backhaul capacity, which reflects the quality of service they receive. Therefore, the primary metric for evaluating the communication constellation is the communication backhaul capacity of the user terminal. Assuming that there are *k* user terminals under the coverage area of satellite *i*, the communication capacity of each user terminal ut is[15]:

$$R^{NG}_{i,ut} = \frac{B}{k}\log_2\left(1 + \frac{P_{gi,ut}}{\sigma^2 + I_{i,ut}}\right) \tag{16}$$

where E represents the available bandwidth of the satellite, $P_{gi,ut}$ represents the transmission power of the satellite, σ^2 represents the additive Gaussian white noise of the satellite, and $I_{i,ut}$ represents the interference between satellite users. For each user terminal *UT*, if there are *m* satellites providing coverage in the area together, the service capacity obtained by the user terminal through satellite collaborative services is

$$CA_{ut} = \sum_{m \in S_{ut}} R^{NG}_{m,ut} \tag{17}$$

As the number of user terminals increases, the total communication return capacity obtained is directly proportional to the number of satellites covering them. The initial constellation is designed using a typical low-orbit polar orbit constellation[16]. Reference (*20*) demonstrates the

coverage advantage of polar orbit constellations in communication through verification, and we consider polar orbit constellations with global multiple coverage. Each orbit is composed of the same number of M satellites, and satellites in adjacent orbits move in the same direction with a phase difference of π/M. The number of N_0 orbital planes and the angular radius φ of the coverage circle satisfy:

$$(N_0-1)\varphi+(N_0+1)\Delta=\pi \tag{18}$$

Where $\Delta=\cos^{-1}[\cos\varphi/\cos(\pi/M)]$, and φ can be obtained via Eq. 19. Therefore, the N_0 orbital planes are separated in terms of angle ϕ, and $\phi=\varphi+\Delta$. The relationships among the number of N_0 orbital planes, the number of satellites M in each orbit, the height of each satellite h, and the minimum elevation angle $\theta_{\min}$ at the user terminal satisfy the following equation[15]:

$$\varphi = \arccos\left(\frac{R_e}{R_e+h}\cos\theta_{\min}\right)-\theta_{\min} \tag{19}$$

$$(N_0+1)\cos^{-1}\left\{\frac{\cos[\cos^{-1}(\frac{R_e\cos\theta_{\min}}{R_e+h})-\theta_{\min}]}{\cos(\frac{\pi}{M})}\right\}+(N_0-1)\left[\cos^{-1}(\frac{R_e\cos\theta_{\min}}{R_e+h})-\theta_{\min}\right]=\pi \tag{20}$$

The design objective of communication constellations is to meet the requirement of satellite coverage while maintaining the minimum elevation angle of users, representing the communication backhaul capacity[21]. At the same time, in order to guarantee the continuity of user communication, the communication constellation requires that the revisit time for users be as small as possible to zero[22]. Typical values of some communication model parameters are presented in Table S1[15]. Among them, the communication frequency band takes the Ka frequency band as an example.

(2) Method of Generating Navigation Constellation

The current global navigation system is a space system situated in a medium to high orbit, which has shortcomings such as low user reception power[23]. At present, low-orbit navigation is one of the technical methods to further improve the navigation performance of satellites. These methods are generally divided into two categories: independent low-orbit navigation and enhanced low-orbit navigation[24-25]. Low-orbit independent navigation is similar to existing global navigation systems, except that the space orbit is designed in low-orbit and has the ability to provide independent navigation services[26]. Low-orbit navigation enhancement is the use of low-orbit constellations to assist existing medium to high orbit satellite navigation systems, enhancing system signal, positioning accuracy, and integrity performance[27].

Considering the availability of existing global navigation systems, the navigation constellation of ISS is mainly used for navigation enhancement, with the main indicator being the available navigation accuracy factor GDOP. Taking the tilted and polar orbits of a Centispace navigation constellation[28] as an example, assume the number of satellites in each orbit is N, the number of orbital planes is P, the phase factor is F, the orbital altitude is h, and the orbital inclination angle is *inc*. When operating in independent mode, only the low-orbit satellite constellation is considered, and the state vector is described as follows[17].

$$\Delta \mathrm{x}=[\Delta \mathrm{r} \;\; c\Delta t_{\mathrm{leo}}] \tag{21}$$

Where Δr represents the user positioning parameter, Δt_{leo} represents the receiver clock delay of the navigation constellation, and *c* is the speed of light. The form of design matrix *H* can be described as follows.

$$H=\begin{bmatrix} \frac{\mathrm{r}_{\text{leo},1}-\mathrm{r}_{\text{user}}}{\left\|\mathrm{r}_{\text{leo},1}-\mathrm{r}_{\text{user}}\right\|} & \frac{\mathrm{r}_{\text{leo},2}-\mathrm{r}_{\text{user}}}{\left\|\mathrm{r}_{\text{leo},2}-\mathrm{r}_{\text{user}}\right\|} & \cdots & \frac{\mathrm{r}_{\text{leo,n}}-\mathrm{r}_{\text{user}}}{\left\|\mathrm{r}_{\text{leo,n}}-\mathrm{r}_{\text{user}}\right\|} \\ 1 & 1 & \cdots & 1 \end{bmatrix}_{4\times n^{\text{nav}}} \tag{22}$$

Where n^{nav} represents the number of visible satellites in the navigation constellation, r_{leo} and r_{user} respectively represent the satellite position vector and user position vector in the geocentric fixed coordinate system. Among them, $\mathrm{r}_{\text{leo},1}$, $\mathrm{r}_{\text{leo},2}$ and $\mathrm{r}_{\text{leo,n}}$ represent different users. Assuming that measurements from different navigation satellites have the same accuracy, the expression formula for dilution of precision (DOP) based on the design matrix *H* is as follows:

$$\text{GDOP}=\sqrt{\text{tr}(H^{\text{T}}H)^{-1}} \tag{23}$$

$$\text{PDOP}=\sqrt{(H^{\text{T}}H)^{-1}_{1,1}+(H^{\text{T}}H)^{-1}_{2,2}+(H^{\text{T}}H)^{-1}_{3,3}} \tag{24}$$

$$\text{TDOP}=\sqrt{(H^{\text{T}}H)^{-1}_{4,4}} \tag{25}$$

The dilution of precision (DOP) reflects the accuracy of satellite navigation and positioning systems, and generally, $\text{GDOP}<5$ can achieve good navigation and positioning performance[24]. The calculation formula for the global GDOP is as follows:

$$\text{GDOP}_{global}=\frac{\sum_{lat=-90^\circ}^{90^\circ}\sum_{lon=-180^\circ}^{180^\circ}\sum_{Epoch=0}^{T}\text{GDOP}(lat,lon,Epoch)}{N_t N_{lon} N_{lat}} \tag{26}$$

For users, it is generally necessary to observe no fewer than four satellites in order to locate, but in practical terms, it generally requires more than six satellites to effectively achieve positioning. Under the same coverage weight, a smaller GDOP indicates better positioning accuracy. According to research from Stanford University, the DOP values of existing satellite navigation systems range from 1 to 3[(23)]. With the addition of low-orbit enhanced constellations, the DOP value is expected to be less than 1.

(3) Method of Generating Remote Sensing Constellation

The design of remote sensing constellations generally considers requirements such as observation coverage area, observation resolution, and revisit time. The coverage area is generally related to the target area. The observation resolution refers to the minimum ground distance that satellites can distinguish, which determines the quality of service of remote sensing constellations. The observation resolution requirement for setting remote sensing constellations is less than r_s meters, and the revisit time is less than r_v seconds.

Assuming that the satellite position is *P*, the orbit altitude is *h*, the Earth radius is R_e, the satellite payload field of view angle is φ, and the ground coverage width is *L*, the formula for calculating the satellite coverage range *L* is:

$$\beta=\arcsin\left(\frac{(h+R_e)}{R_e}\sin(\varphi)\right) \tag{27}$$

$$L = 2R_e \cdot (\beta - \varphi) \tag{28}$$

$$E_{rsl} = \frac{\theta h}{\cos\theta} \tag{29}$$

Where L is the coverage area of a single satellite on the Earth, and θ is determined by the size of the satellite's Earth observation sensor and the size of the imaging element matrix. The performance of the observation is related to the sensor components. To achieve global single coverage, the total coverage area of all satellites in a constellation is generally twice the total ground area[20]. Therefore the coverage area of remote sensing payloads needs to achieve twice the coverage.

Remote sensing satellites mainly include electronic reconnaissance and optical imaging. The width of electronic reconnaissance satellites can reach 2000-3000 kilometers, but the accuracy is only 10-30 kilometers, while the optical width is only 30-60 kilometers; however, the spatial resolution accuracy can reach 0.1-2 meters, and the values are related to the orbital altitude[14,29]. The resolution of Earth observation payload sensors is also related to the number of satellites. The more satellites there are, the shorter the revisit period may be, while the lower the orbit is, the higher the resolution can be obtained. Therefore, this approach also involves optimizing the number of satellites and orbital heights.

2.3 Basic Scheme Generation Algorithm

Based on the set of task requirement inputs, we adopt a hierarchical collaborative multidisciplinary optimization method[30] for the design of the basic constellation. During the algorithm design process, the system level design sequentially checks the feasibility of the input design scheme. The optimizer for each discipline will pass through shared variables with optimizers from other disciplines during optimization to achieve fast solving. The system-level optimizer will iteratively interact with the subsystem-level discipline optimizer to obtain a preliminary constellation that belongs to excessive design[31].

On the premise of ensuring service performance, in order to achieve the lowest cost and maximum sustainability of ISS, the standard for removing satellites in the deployment optimization process of ISS satellites and sensors is: sensors are sequentially placed on the satellite cluster visible to the corresponding coverage grid. The change in the service performance of resource pools after computing mobile sensors. After moving all the satellites, the improvement in service performance of the resource pool caused by the change in the position of each sensor is ranked. If there is one that is better than before and meets the service requirements, choose the optimal location as the new sensor deployment location. In the process of optimizing deployment, the limit on the number of sensors deployed per satellite can be increased. After looping through all sensors, the satellites without sensors are removed.

A space system is a space network that changes over time and has spatial attributes. Therefore, when optimizing, cycle time and grid partitioning are needed. The sensors carried by each satellite are recorded using a symbol matrix, and the dimensions are the number of sensor types and the number of system satellites. The number of sensors carried by each satellite is limited by its volume, power consumption, and processing capacity. In the process of optimizing sensor deployment, this paper currently considers that each satellite has communication and another type of sensor. It is also allowed to carry three types of loads. However, considering the current resource capacity of satellites, two types of sensors are limited.

During the removal process, the removal of navigation sensors is different from that of remote sensing sensors. In the new deployment location, if the navigation resource GDOP

value in the resource pool is higher than the original deployment location, the new deployment location can be adopted. It should be noted that the limitation on the number of sensors deployed on each satellite should also be considered.

The iSat constellation of an ISS does not mean that the architecture of the ISS is only applicable to that constellation, while other constellation configurations are not applicable. The openness of the ISS architecture allows it to be applicable to other constellations. The purpose of designing an iSat constellation is to demonstrate that, under the constraints of an ISS architecture, a constellation can reduce the number of satellites in the entire space system, optimize space orbit resources, improve spatial sustainability, and provide even better service and coverage performance than existing space systems.

The design of the iSat constellation aims to optimize the orbital density of the space orbit, based on meeting the global demand for space resource services such as navigation, communication, and remote sensing, and is optimized by a basic constellation. A basic constellation is a constellation that uniformly covers the world, optimizing the number of satellites and deployment positions of sensors based on this constellation.

Fig. S7 shows the optimization based on the OneWeb constellation, which has 720 satellites[15]. Under the constraint of at least 3 layers of coverage and 95% coverage, the number of constellation satellites optimized using the iSat constellation design optimization method is 450, a reduction of by approximately 37%.

2.4 Multiple resource coverage based on satellite cluster

Due to the limitation of volume and power consumption, satellites of intelligent space-based systems generally do not carry many kinds of sensors, which not only puts forward high requirements for satellite integration technology, but also increases the cost of building intelligent space-based systems as infrastructure due to multiple resource coverage, which is actually a waste of sensor resources. Therefore, it is necessary to design requirements to optimize the deployment of satellites and sensors. The basic principle of optimization is to eliminate the redundant resource coverage of the target area, as shown in Fig. S6(a). Assuming that the service demand of the target area only requires the double coverage of such resources, the four satellites in the figure can share resources, so the resource coverage is redundant for users, and the sensors of satellites 2 and 4 can be removed.

To solve the above optimization problems, this paper proposes an optimization method of satellite and sensor deployment based on resource coverage. According to the number of coverage layers, coverage rate and service capacity requirements of a given system, then the ground area is gridded. For each area grid, the satellite set visible to the area is first found. Then, the satellite set is used as a resource pool for cross domain fusion to fuse and eliminate satellites and sensors, so as to optimize the number of satellites and load deployment under the constraint of meeting service requirements. This step can be carried out for each time slot segment of the space-based system. The longer the search time is, the more accurate the optimization result is. However, there may be a problem that the optimization time is too long. The multiple time of the satellite cycle can be selected.

2.5 Service Mode

ISS can provide space resource services for individuals, regions, countries and even the whole world. For individuals, satellite services can be customized in the same way as DiDi and Uber. Satellites are used over different countries or regions in the form of lease. It can also play the role of communication emergency service in disaster monitoring and international search and rescue, as shown in Fig. S14. This paradigm enables all humanity to

jointly build a global space system, playing an important role in global scientific research, global governance, and other aspects.

In addition to reducing the number of satellites and bringing benefits to the sustainable development of the space environment, ISS not only have the service capabilities of traditional space systems, but also can provide flexibly defined and enhanced services on demand in the form of task clusters. The cooperation of multiple satellites can enhance the service capability of certain aspects of space resources, or provide various services[32]. For example, cooperative remote sensing of multiple satellites can improve the area ratio of the target area. If a large-scale regional disaster occurs in a certain area, using sensors from multiple satellites to detect the situation in the disaster area can facilitate the rapid dissemination of disaster information and emergency decision-making (Fig. S14e). Another example is satellite cooperative communication, which can improve the communication rate and bandwidth (Fig. S14c). Collaborative navigation enhancement can enhance the navigation service capability and expand the coverage of the target area through satellite collaboration and reconstruction functions. In our Live-Virtual-Constructive (LVC) experiment, the navigation horizontal DOP value can be increased to 0.0127, and the vertical DOP value can be increased to 2.4451. The received power of the integrated signal fluctuates between 19dB and 45dB. When there is strong interference in the received power of the integrated signal, the carrier to noise ratio can still be 45dB and the positioning is normal. In the part 6 of the supplementary materials, an example will show the enhanced service capability of ISS on demand.

3. Satellite Number Estimation

Based on data from Union of Concerned Scientists (UCS), the United Nations Office for Outer Space Affairs (UNOOSA), and other sources, polynomial fitting predicts satellite numbers. First, according to UCS[37] and UNOOSA[38], the number of satellites launched or in orbit each year from 2008 to 2023 is obtained, as shown in Table S2 and Table S3.

Because the number of satellites regularly released by the UCS is only updated until May 1, 2023, the satellite data for 2030 were obtained from references (39) and (40), respectively. According to the prediction given in reference (41), there are 100000 satellites. Based on the satellite data from 2008 to 2022 and 2030 mentioned above, we performed a polynomial fitting using the least squares method with a degree of 3, and obtained a polynomial as shown in Eq. 30.

$$f(n) = 31.9747 \cdot n^3 - 1.9329 \cdot 10^5 \cdot n^2 + 3.8947 \cdot 10^8 \cdot n - 2.6159 \cdot 10^{11} \tag{30}$$

If we follow the current development model and repeat the construction of independent space systems with different functions[42], the number of satellites in the future space environment will be the same as the space systems proposed based on the current stage in Fig. S8. By 2030, the number of satellites can reach 100000, while by 2036, the number may reach 270000. According to a research report released by the US Government Accountability Office, it is estimated that by 2030, the global number of satellites could reach approximately 58000 (43). As shown in Fig. S8. Assuming that the ISS architecture based on the cloud-pool-terminal (CPT) model proposed in this article is adopted to build a space system, according to its resource sharing and on-demand service characteristics, countries around the world do not need to repeatedly build the same space system. Like the Internet, countries around the world jointly build and use the same space system. After continuously supplementing and improving the global space infrastructure, humans only need to immediately replace and upgrade failed satellites, which can avoid unlimited growth of satellites and ultimately break through orbital

space capacity, causing irreversible harm. According to the statistics in reference (44), the three planned giant constellations under construction adopt the CPT mode, and the satellites adopt an intelligent satellite architecture. Therefore, by 2030, the number of satellites will only reach 33714, and after 2036, this number will stabilize to about 48000. Based on the above data and using the least squares method for polynomial fitting, with a degree of 3, the obtained polynomial is shown in (31).

$$f(n) = 1.8058 \cdot n^3 - 1.0844 \cdot 10^4 \cdot n^2 + 2.1704 \cdot 10^7 \cdot n - 1.4481 \cdot 10^{10} \tag{31}$$

Based on these fitting data, the number of satellites in 2036 will only reach 48000, as shown in the predicted data in Fig. S8. Compared to the orbital space capacity of 175000 satellites, open and shared sustainable mega-constellation (OSSMC) has reduced the number of satellites by 72.6%. Compared to the existing one million satellite applications, the number of satellites in OSSMC is only 1/20. And it can maintain this level basically unchanged, while also meeting service needs.

Although increasing the number of satellites can improve the service performance in a certain range, considering the diversified needs of users, the sustainable development of space and the cost of system construction, simply increasing the number of satellites is not the best solution. For example, in the navigation service, according to the research in reference (33), when the number of satellites is 1584, the GDOP value is about 0.7 between 35 and 55 N. After the number of satellites increased to 4,408, the GDOP in this area dropped below 0.5. The impact of continuing to increase the number of satellites on GDOP improvement tends to be flat. The same applies to communication services. References (34), (35) and (36) use different communication models for different application scenarios, but the final conclusion shows that the user communication capacity does not increase linearly with the increase of the number of satellites. When the number reaches a certain level, the communication interference between users will increase significantly. This is consistent with our research, that is, large-scale deployment of satellites on a global scale is not an efficient and sustainable road.

4. System Cost Estimation

Fig. S9 illustrates the cost decomposition structure of the satellite cost model used in the paper, which is based on the reference model proposed in (*11*,*45*). Among them, nonrecurring expenses, such as research and development funds and ground construction costs, are marked with a white triangle in the bottom right corner of each box, with the letter "NR". Recurrent costs, which are the costs incurred for each unit constructed or paid as operating expenses annually, are marked with a black triangle with the letter "R", without considering the cost of ground segments[47].

The cost model of satellites can be divided into five categories[11], namely BOMS, sensors, launch, structure, and program costs. Among them, BOMS is decomposed into different subsystem costs, and the program part also includes system engineering and management. The parameter cost model of satellites uses the SSCM model[48], while the small satellite cost model was developed by aerospace companies to predict the development cost of modern small satellites (below 1000 kilograms). These models are based on cost estimation relationships (CERs) and provide cost estimates for the mass (or other parameters) of the stator system. SSCM provides a comprehensive estimate of nonrecurring costs plus first unit costs (assuming nonrecurring costs account for 60% of this value and first unit costs account for 40%)[48].

The structure, thermal control, attitude determination and control system (ADCS), electrical power system (EPS), telemetry, tracking and command (TT&C), mass of the sensor and propulsion are $m_1, m_2, m_3, m_4, m_5, m_6, m_7$ respectively, in kilograms. The following models are used as shown in Table S4. Based on this model, the cost estimation formula for intelligent satellites is as follows:

$$C^{BOMS} = 355 + 5.7m_2^2 + 1850 + 11.7m_3^2 + 486 + 55.5m_5^{1.25} + C^{onboard} \tag{32}$$

$$C^{Structure} = 407 + 19.3m_1 \log_{10}(m_1) \tag{33}$$

$$C^{Launch} = 0.061 \cdot C^{BOMS} + 0.245(m_7 / 0.4536)^{0.667} \tag{34}$$

$$C^{Program} = (0.139 + 0.229) \cdot C^{BOMS} = 0.368 \cdot C^{BOMS} \tag{35}$$

$$C^{onboard} = 0.563 \cdot (m_6 / 0.4536)^{0.762} \tag{36}$$

$$C^{Sensor} = \sum_i C_i^{sensor} = \sum_i 0.305 \cdot ((\frac{m_i^{sensor}}{0.4536}))^{0.477} \cdot P_i^{0.139} \cdot (\frac{DR_i}{1000})^{0.049} \cdot P_i^{0.139} \cdot (\frac{DR_i}{1000})^{0.049} \tag{37}$$

Reference paper on sensors (*19*). Among them, m_i is the mass of the sensor (kg), P_i is the working power of the sensor (W), and *DR* is the downlink data rate of the sensor (bits/s).

The system cost calculation results are shown in Fig. S13. In the cost estimation of a single satellite, Hawkeye 360 satellite has the highest cost, followed by Centispace satellite, and finally Starlink satellite and iSat satellite. The cost configuration of iSat satellite and Starlink satellite is the same, while in the cost estimation of the whole space system, Starlink system is the highest because it has the largest number of satellites. By comparing the cost of Starlink system and ISS and the cost of each subproject, it can be seen that the system cost of ISS is reduced by 16.68% compared with Starlink system, while compared with the combined system, the cost is reduced by 47.88%. This shows that the ISS can effectively reduce the system cost while maintaining the service quality close to Starlink system and combined system. From the perspective of cost subprojects, the cost of ISS in terms of sensors is higher than others. On the one hand, it reveals the reasons for improving service quality but reducing costs. On the other hand, it also shows that the ISS architecture based on CPT can maximize resource benefits.

5. Sustainable Gain Calculation

Space orbital density, space volume collision probability (SVCP), and orbit impact score (OIS) are defined to evaluate the impact on the space environment. SVCP measures the crowding of orbit resources, and OIS quantifies the impact of space missions on orbital resources, considering both current and future spacecraft deployment.

1. Space Orbital Density and Space Volume Collision Probability

The satellite orbit density is defined as the ratio of the number of satellites to the volume of space. Divide the number of satellites in the orbit by the corresponding volume on a scale of 50 km. Note that it is a three-dimensional space.

$$\rho_h = \frac{\Delta n_h}{\Delta V_h} \tag{38}$$

The collision probability in sustainable rating models is closely related to the satellite orbit density[49]. The mission index module of space sustainability rating is directly derived from the

European Space Agency's debris index[50], which quantifies the degree of harmful physical interference that space missions may cause to the space environment. The paper also considers space debris data, transforming space orbit density into consideration of SVCP. SVCP evaluates the degree of crowding of space orbit resources and characterizes the probability of collisions with other objects. The more satellites deployed in the orbit, the larger the SVCP. If it reaches a certain level, that is, space is filled with satellites or other objects, humans may no longer be able to enter space. The definition of SVCP is as follows:

$$I_{SVCP} = \rho_I (2\rho_I + \rho_D) \times 10^{16} \quad (39)$$

Where ρ_I is the density of satellites or other spacecraft, ρ_D is the density of space debris, which is calculated via the simulation method of gas dynamics theory. The debris density *p* and flux are extracted from the ESA software MASTER[51], and the cross-sectional area of the considered object and the selected time period are used as inputs[52].

2. Orbit Impact Score

The orbit impact score (OIS) is used to calculate the degree of impact of space missions or systems on the space environment, in order to evaluate the degree of degradation of orbital resources[53]. The OIS quantifies the severity of potential debris based on the increase in the collision probability of operating satellites. It is judged from two perspectives: one is from the inside out perspective, represented by the severity coefficient (SF), and the other is from the outside in perspective, using the exposure coefficient (XF) to evaluate the degree of damage to the functional value of rail resources. It not only calculates the distribution of objects in the current space orbit environment, but also considers the impact of spacecraft in the future. This is because the impact of spacecraft deployment in orbit is persistent, as without human intervention, they will remain in space orbit for a long period of time, occupying orbital resources[54]. For future predictions, NASA's decomposition model is used to simulate the impact of fragmentation[55]; then, a representative set of operating satellite groups is defined, and the collision probability of these objects due to simulated fragments is calculated. The definition of *OIS* is as follows[53]:

$$OIS = A_c \cdot k \cdot (M)^{0.75} \sum_{i}^{orbits} t_i \cdot CF_i \quad (40)$$

The *CF* is the characteristic factor of a given orbit, which is the product of the exposure level factor *XF* and the severity coefficient *SF*, i.e.:

$$CF_i = XF_i \cdot SF_i \quad (41)$$

The definitions of the exposure level *XF* and severity coefficient SF are as follows (*56*):

$$XF_i = \bar{\phi}_{h,inc,t} [\#, m^{-2}.yr^{-1}] \quad (42)$$

$$SF_t = \int_0^t e^{-\frac{t}{\rho(th)}} \quad (43)$$

$$\rho(th) = e^{-\frac{t}{128.3 - 0.585892h + 0.00067h^2}} \quad (44)$$

Equation (44) represents the space debris flux at a given height *h*, inclination angle *inc*, and time *t*, expressed in units.

This paper evaluates the impact parameters of the above four systems on the space environment, mainly considering the object density of the space orbit, the space volume collision rate (SVCP), and the impact score OIS of the space mission on the orbit resources after the deployment of space systems. The parameters and calculation results used are shown in Table

S5. Fig. S16 show the OIS for four space systems and three scenarios. It can be seen that the space volume collision rate of ISS is reduced by 28.7% compared with the combination system, nearly more than a quarter. The OIS of ISS decreased by 53.15%, more than half.

The gain analysis above is only applicable to a small single-layer space system. Fig. S8 shows the number of satellites in three scenarios, namely GAO estimation, estimation based on the existing application constellation, and estimation based on CPT paradigm. If the space systems with independent functions are constructed repeatedly according to the current development mode, the number of satellites contained in the future space environment will be the same as the space systems proposed at the present stage. The number of satellites would reach 175000 around 2030 to 2036, which will exceed the safety limit. On the contrary, because the CPT paradigm's characteristics of resource sharing and on-demand service, countries around the world can dynamically configure the satellites above to provide services for themselves. The ISS are space infrastructures serving each country all the world like the Internet. Countries around the world jointly build and use one space system. After continuously replenishing and improving the global space infrastructure, humans only need to replace and upgrade the damaged satellites in time, which can avoid the unlimited growth of satellites and finally break through the orbital space capacity, bringing irreversible harm. At this time, according to the forecast data shown in Fig. S8, the number of satellites will be stable at around 48000 after 2036, which can keep the level basically unchanged and meet the service demand.

6. System Service Capability in Semi-Physical Simulation Experiment

The study implemented semi-physical experiments on the CPT architecture for intelligent space systems, simulating a 450-satellite ISS constellation on a cloud server with real-world drone-mapped satellites. It evaluated mission effectiveness through task-oriented, on-demand services, contrasting traditional resource isolation with ISS's shared resource pools. Tasks were randomly assigned, and metrics such as response time, resource utilization, and success rates were analyzed. The CPT paradigm, utilizing resource sharing via methods like combination and migration, demonstrated significantly enhanced task completion probability and service quality by optimizing resource paths, exemplified by the Edmond Karp algorithm. The intelligent space-based system completes communication, navigation, and remote sensing tasks proposed by users by configuring satellite clusters. The terminal is used to propose tasks to the cloud or satellite and receive results. The three scenarios are emergency communication in international search and rescue, navigation enhancement in interference environments, and wide area satellite collaborative remote sensing. This underscores ISS's superiority in dynamic resource management and collaborative task execution.

1. Scene diagram

We use 5 drones each carrying satellite hardware as real satellites to simulate satellite in orbit motion. The remaining satellite movements and their services are simulated and run by cloud servers through containers.

2. Navigation:

When existing navigation positioning is interfered with, low orbit satellites can be configured as navigation enhancement functions to provide enhanced navigation signals for ground users.

3. Communication:

A measurement and communication integrated system is adopted between satellites, and communication is the basis for satellites to share and cooperate with each other. Data can be

transmitted during measurement. In this way, during international search and rescue operations, satellite emergency transmission of partial data can be configured.

4. Remote sensing:

In the event of a wide area natural disaster, such as an earthquake, satellites can simultaneously activate remote sensing functions and transmit multiple regional images, which can obtain remote sensing information with a larger coverage area.

Taking collaborative enhancement of the transmission signal power as an example[57], when a certain area needs to receive high-power signals to achieve anti-interference, but low-orbit satellites may not have the ability to transmit higher-power signals than the goal satellite, several satellites can work together to improve the quality of the transmission signals. Assuming that the target receives signal power P_{signal}, d is the spatial distance between the iSat satellite and the target, P_{iSat} the design value of the power of a single iSat satellite, and $N_{\min}$ is the number of iSat satellites participating in collaboration. To ensure service quality, a power margin of 3-5 dB is set, which is 3 times the received signal power[58].

$$P_{receive} = 3P_{signal} \tag{45}$$

According to the free space propagation formula

$$P_T = P_R - G_T - G_R - 20\lg\left(\frac{\lambda}{4\pi d}\right) + L_A \tag{46}$$

By substituting $P_R = P_{receive}$, the total power P_T required for the iSat satellite to transmit and receive signals P_T can be calculated based on the distance between the iSat and the target, as well as the gain of the antenna. When N iSats are used to synthesize signals, the signal-to-noise ratio gain is N^2 times[59]. Therefore, assuming that the power design value of a single iSat is P_{iSat}, the minimum number of satellites required can be calculated

$$N_{\min} = \left\lceil \sqrt{\frac{P_T}{P_{\mathrm{iSat}}}} \right\rceil \tag{47}$$

The corresponding situations for the two signals mentioned above are calculated, namely one 5 W and one 10000 W. Assuming a satellite altitude of 650 km and a single satellite power of 50 watts, the required number of iSat satellites is calculated as shown in the Table S6 below, with a carrier frequency of 5 GHz [(60)].

7. Supplementary Analysis of System Service Performance

This section will compare the service capabilities of ISS with existing systems in communication, navigation, and remote sensing. Through the comparison in this section, how the new paradigm based on CPT can achieve the service capabilities of multiple traditional space systems with one space system will be illustrated.

7.1 System Performance

1．ISS, Starlink, and Iridium II

审图号：GS 京（2025）1735 号

Fig. S10 evaluates the relationship between the communication service capacity of the designed constellation and the coverage multiplicity and user density in the optimization objectives. It is evident that Starlink has a larger capacity due to doubling the number of

satellites[15]. As the user density increases, the service capacity (i.e. available bandwidth) allocated to each user also decreases. Because as the number of users increases, there will be more interference links in the satellite ground link. However, this capacity is the sum of the available capacities of each satellite, and in reality, each satellite is distributed in different orbits, and the actual available capacity varies[45]. Moreover, under the collaborative and shared working mode of the iSat constellation, it can be met by configuring and scheduling resources when users demand higher capacity. This experiment will be reflected in task effectiveness.

From 审图号：GS 京（2025）1735 号

Fig. S10, it can be seen that the coverage of users by the ISS and Starlink systems is the same, but due to the fact that Starlink has twice as many satellites as ISS, the average communication capacity of users is higher than that of ISS. In order to compare with the large number of Starlink, the communication capacity of ISS has been proportionally enlarged based on the number of Starlink and ISS[46]. At this point, the user communication capacity of the two systems is equivalent. However, low-orbit communication constellations are generally used to serve remote areas, which belong to sparsely populated areas, while densely populated areas can be replaced by ground networks[61]. Therefore, the communication capacity of ISS can be allocated to sparsely populated areas as needed, which can achieve the same service capacity as Starlink in sparsely populated areas.

2. ISS and Centispace

Centispace belongs to the low-orbit navigation enhancement constellation[28]. Because low-orbit navigation enhancement constellations are generally used as auxiliary enhancement systems for satellite navigation systems[17], the Beidou system 3 (BDS-3) is added to the Centispace and ISS respectively when comparing performance, and the GDOP values of the systems are evaluated. The results are shown in Table S7, which shows the global GDOP values of the two system schemes and the minimum, maximum, and average improvement percentages of the GDOP values of the BDS-3 by the two low-orbit satellite systems. After addition of low-orbit satellite systems, there has been varying degrees of improvement in the GDOP values of BDS Among them, the improvement of GDOP value by ISS is significantly better than that of the Centispace, with an average improvement of 51.07%, which is 19.05% higher than that of Centispace.

3. ISS and Hawkeye 360

Fig. S12 shows the heatmap of the earth coverage weights of the Hakweye 360 and ISS. Due to the limited number of Hakweye 360 satellites, the coverage area is limited and there are significant gaps. And the coverage weight is only two layers or more in the key areas. The ISS can achieve global coverage with a minimum of 15 multiplicities. It can be seen that in remote sensing, the ISS is significantly stronger than the Hakweye 360.

7.2 Task Effectiveness Simulation

Unlike traditional space systems that are based on functional services, ISS provide task-oriented and on-demand services, therefore simulation analysis of the mission effectiveness of space systems need to be worked in different paradigms. To control variables, the ISS is set to provide the same communication, navigation, and remote sensing task types as traditional space systems, and the required computing, storage, sensing, and other resource requirements for the task are configured. The task is randomly assigned, and the results of task response time, resource utilization, and task success rate during the simulation process are collected and statistically analyzed. The scenario is shown in Fig. S15. Based on the set constellation, corresponding task types are set to simulate satellite reception and task execution[62]. Based on

this, the spatiotemporal, network, and other relationships of the space system in orbit can be simulated. Users can randomly propose requirements, satellite reception tasks, and perform analysis and functional execution. The satellite constellation parameters used in the task simulation are shown in Table S8. It should be noted that due to the large number of satellites in Starlink, the task simulation is difficult to operate normally. Therefore, we referred to the number of OneWeb's constellation parameters and only used 720 satellites in Starlink. The main parameters of the set task type are considered as shown in Table S9.

In ISS, satellites share resources with each other through resource pools and collaborate in processing and executing tasks, providing possibilities for improving resource utilization and service efficiency. The methods of resource sharing can include combination, migration, etc., as shown in Fig. S17. *Vob* and *vdc* represent the virtual starting point and virtual endpoint, respectively. *SSr*, *Sdr*, *CR*, *Sto* and *NS* represent signal sensing, detection sensing, computing, storage, and network resources, respectively, while *O* and *D* represent the user and target endpoints, respectively. Under the CPT paradigm, in order to complete the autonomous detection, processing, and transmission tasks of the target area, from *VOB* to *VDC,* resource pooling provides a lot of choices. We only need to choose the optimal combination of resources from them to achieve the best service quality.

When the space system is in traditional mode, due to resource isolation, it can only be processed locally and cannot be shared. Because the target is not visible, there are no resources available to complete the task within this time slot, meaning that *VOB* to *VDC* are not connected. Until the 24th time slot, there will be satellites visible to the target area, so there is a resource path to complete the task. Fig. S17c shows the operation of the ISS in the CPT paradigm, where the shortest path is the resource path found based on the Edmond Karp shortest path algorithm[63] to meet the task requirements. Obviously, compared to the traditional mode where each space system functions independently, the CPT paradigm greatly increases the probability of task completion, and by optimizing resource paths, it can also improve service quality.

7.3 Task Efficiency Analysis

1. Average Response Time and Average Waiting Time

Fig. S18 shows the average response time of the same space system to complete tasks in the two paradigms. The graph shows that in both modes, the task response time increases with an increase in the number of tasks. In the traditional mode, the average response time of the task is approximately 9.28 seconds. Even if the number of tasks is small, the response time is too long because tasks can only be processed locally, approximately 11.49 seconds. In the "cloud pool side" paradigm, when the number of tasks is small, they can be executed quickly without waiting for too long, with a time of approximately 6.63 seconds. It is worth noting that when the number of tasks increases by more than 180, the average waiting time decreases. This is because the success rate of tasks has increased, while the failure rate of delayed tasks has decreased, which will be analyzed in the next section. In contrast, there are fewer waiting tasks in the queue, resulting in a decrease in the average waiting time. The average response time of the last two paradigms tends to be the same because when the workload reaches the cluster load, it will enter a balanced state.

The result of the average waiting time is shown in Fig. S18b. The average waiting time for tasks in the traditional mode is 17.84 seconds. Even if the number of tasks is small, the waiting time is long because it can only be processed locally and the resource waiting time is long. In the CPT paradigm, when the number of tasks is small, they can be executed quickly without waiting

for too long. However, as the number of tasks increases, it takes more time to wait for sending and receiving tasks, which is approximately twice the average waiting time of the tradition mode.

2. Task Success Rate and Task Failure Rate

In terms of the task success rate, Fig. S19a shows that through resource sharing, the CPT paradigm can greatly improve the task success rate of space systems, increasing from 26.21% in the traditional mode to 45.73%, an increase of 19.52%. This is because resource sharing allows more tasks to be allocated resources, while tasks in traditional models must wait for resource allocation. When there are few tasks, the success rate of the task will undergo significant changes. This is because few tasks and resource requirements are easily met. However, due to visibility and satellite movement, some tasks may fail, resulting in significant fluctuations in task failure rates when tasks are scarce.

Compared to the task success rate, the task failure rate is the ratio of the number of failed tasks to the total number of initiated tasks during the experimental period. This article summarizes the failure rate of tasks due to mobility, insufficient resources, and unsatisfactory latency. Fig. S19b shows that the CPT paradigm can share resources, which solves the problem of tasks failure without increasing resource satisfaction. Therefore, the task failure rate decreased from 51.45% (peak 75.00%) in the traditional mode to 1.64% (peak 8.57%). However, resource sharing brings other issues. The first is delay. The CPT paradigm requires the transmission of information, migration tasks, transmission of information, and application software with other satellites, all of which can cause task delays. Tasks with high real-time requirements may lead to task failure. Therefore, in the CPT paradigm, task failures caused by delays are higher than that in the tradition mode. The task failure caused by delay in traditional mode is stable at around 25.19% in Fig. S19b. Due to its local processing, only the local task queue will cause delays. The reason for the decrease in the task failure rate under the CPT paradigm is that when there are fewer tasks, task planning algorithms pursue resource load balancing of the system and allocate tasks as much as possible in the resource pool, which may lead to some tasks failing with a probability of 57.40%.

In addition to the two issues mentioned above that may lead to task failure, mobility is also a key factor. The mission failure caused by satellite mobility refers to the situation where the satellite leaves the target area due to its orbital motion or is not visible to other satellites. As shown in Fig. S19b, this proportion is very small, at only 3%. At the same time, it can be observed that the CPT paradigm has a higher failure rate due to mobility compared to the tradition mode. The reason for analyzing data is that data transmission in resource sharing increases the probability of latency and invisibility. In the traditional mode, the task failure rate is relatively low because the task is only sent to satellites visible to the target and is only executed locally.

3. Resource Utilization Rate

The paper calculates the energy utilization rate and network utilization rate for each mode, as shown in Fig. S20. The average bandwidth allocated to the task and the network resource usage in each experiment were also calculated. As shown in the Fig. S20, the average allocated bandwidth of a task decreases as the number of tasks increases. The use of network resources in the CPT paradigm increases with the increase of tasks. The reason why the tradition mode consumes fewer network resources is that there is no resource sharing between satellites, and information is not transmitted between satellites. In the CPT paradigm, when the number of tasks is small, network resources are used less because resources are still abundant and do not require a large amount of information transmission and task migration. As the number of tasks increases,

the consumption of network resources will rapidly increase due to the need for continuous transmission of information, sharing of resources, and completion of tasks.

The energy consumption situation is shown in Fig. S20b. It can be observed that when the task volume is low, the system overhead required for maintaining resource pools in the CPT paradigm leads to higher network resource utilization. Regardless of the paradigm, the more tasks involved in execution, the higher the average energy consumption of the system. However, when it increases to 180 tasks in the traditional mode, it does not increase, indicating that the peak of task execution in this mode has been reached. In the traditional mode, due to the lack of data transmission in the system, the average energy consumption is the lowest in comparison. With the increase in energy consumption caused by data transmission, the consumption rate of the CPT paradigm slows down and eventually tends to stabilize, with the traditional mode accounting for 26.12% and the CPT paradigm accounting for 39.88%. For satellites, it is worthwhile to increase the success rate of tasks brought about by energy consumption and network usage. Because satellites can be charged with solar energy, it is very cost-effective to consume energy to obtain better services without affecting satellite health.

From the above results, it can be seen that the CPT paradigm has improved the success rate of tasks while maintaining basic service performance. This is crucial in scenarios where high task success rates are required. If the power is sufficient and the task is not urgent, the working mode can be switched. The working mode should depend on the needs of the task, rather than remaining unchanged to achieve optimal service performance, which also reflects the precise service characteristics of ISS.

7.4 Space User Connectivity

ISS not only serves ground users, but also provides service support for space users. Due to the flexible definition of ISS functions, providing services for space use is more advantageous. In order to conduct a brief analysis of the performance in this area, we referred to reference 64 and set up two typical space users. These two are the MENGTIAN of the Chinese space station and a sun-synchronous orbit satellite. These two users can be seen in Fig. S21(b). Their parameters are:

(1)The MENGTIAN of the China Space Station (CSS), at a 400km altitude, 51.6 degree inclination.

(2)An earth observation satellite in sun-synchronous orbit (EOSS) at an altitude of 500km.

Mega-constellations are generally thought to have a satellite number over 1000. Therefore, under the same design process, in order to support more space users, we further expanded the number of satellites on the ISS and obtained another ISS (eISS) with 1320 satellites. The system is shown in Fig. S21(a). Its orbit altitude is 800km, 1000km, and 1200km. In addition, in order to maintain consistency with the previous analysis, the ISS of 450 satellites will also be compared and analyzed together. We will give the service performance of both systems for space users.

Firstly, we conducted an analysis on Doppler frequency shift and frequency shift rate, satellite availability and duration for space users. The results of Doppler frequency offset and frequency shift are shown in Fig. S22. As shown in the figure, for space users, the elevation angle has a significant impact on Doppler. When the Doppler shift is large, due to the availability of multiple satellites, satellite switching can be used to maintain service quality. In terms of altitude, due to the fixed height of the ISS constellation, as the user's orbital altitude increases, the distance from the ISS decreases and the Doppler frequency offset decreases.

The results of space connectivity for CSS and EOSS can be seen in Fig. S23 to Fig. S25. Based on the analysis of satellite availability in Fig. S23 to Fig. S25, ISS can provide good connectivity for space users. From the number of satellites that can provide connectivity

services, the minimum number of satellites that can provide connectivity services for CSS and EOSS is 60 or more. But in reality, it definitely involves issues such as multi-user and satellite allocation. From these three graphs, it can be intuitively seen that the more quantity, the stronger the connectivity. When ISS only has 450 satellites, the number of connected satellites and available time are already very large. It can be found that the number and duration of available satellites for these two users are significantly higher than those for ground users. This is because both space users and ISS are working in space orbit. That is to say, no matter where space users operate, ISS has some satellites visible to them, which can provide them with customized services. Of course, this also requires support from space users in terms of communication interfaces and network protocols.

In order to analyze the number of visible satellites on the ISS by ground users, we selected four ground stations within the target area for observing the ISS. The coordinates of the four ground stations are shown in the Table S10, and the observation results are shown in Fig. S26. A minimum elevation angle of 25 degrees was used for all visibility calculations. This threshold accounts for significant tropospheric signal attenuation, potential local terrain masking near the horizon, and reduced antenna gain at very low elevations[64]. Therefore, we no longer separately consider the impact of horizon masking and station latitude. From Fig. S26, it can be seen that the four ground stations in the target area can maintain a visible satellite count of at least 30 for ISS. This is consistent with the coverage conclusion obtained in Chapter 7 of the paper. Meanwhile, comparing the simulation duration of 6 hours and 24 hours, it can be observed that the number of satellites exhibits fluctuations. Within a day, ground stations with lower dimensions have fewer visible satellites than those with higher dimensions.

Fig. S27 and Fig. S28 are both about the visible time of the ground station to each satellite. Fig. S27 and Fig. S28 show the visible duration of the ground station to the satellite. From Fig. S27 and Fig. S28, it can be observed that due to the high dynamism of low Earth orbit satellites, their return time is relatively fast. The ISS repeats the coverage characteristics of the target area twice within a day. From the duration of Fig. S29 and Fig. S30, it can be seen that most satellites can serve ground stations for half an hour within a day.

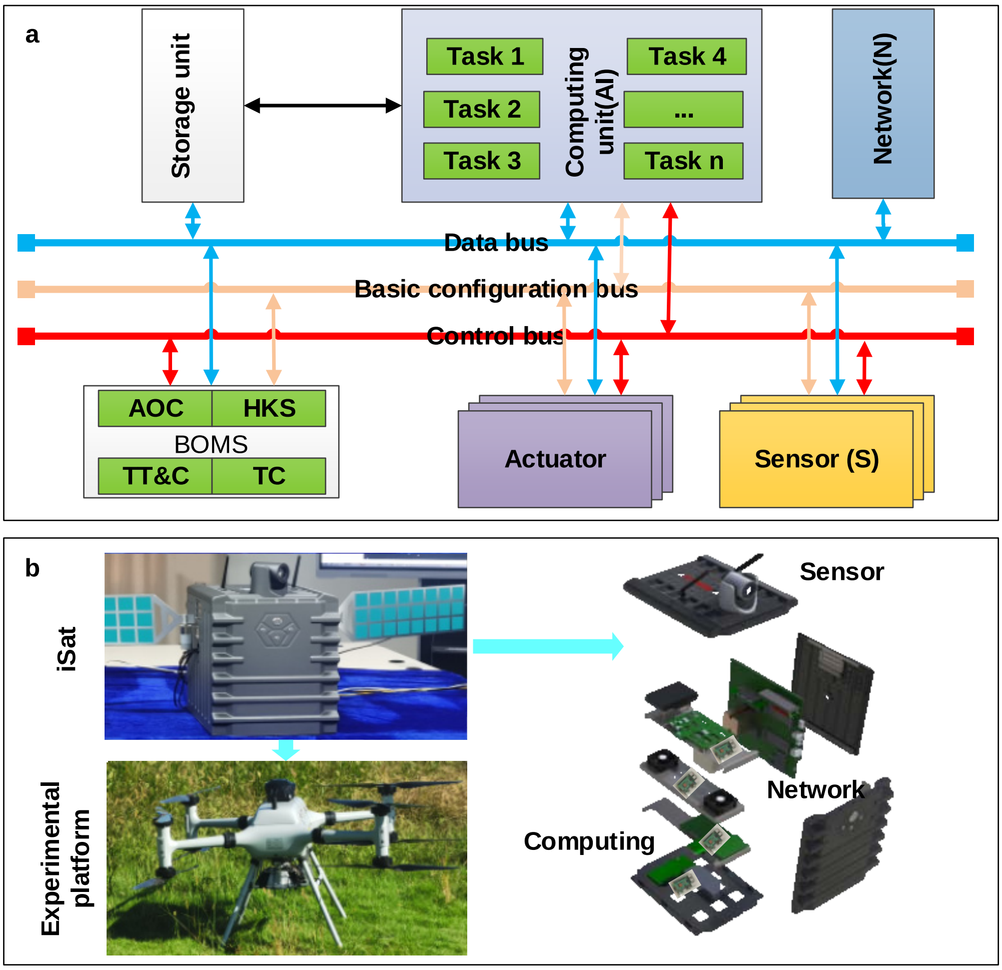

Fig. S1

ISat hardware architecture and experimental platform. a, iSat's SNAI architecture. Each module is connected to each other through a bus, which is divided into three types: data bus, basic configuration bus, and control bus, respectively, to complete the functions of data transmission, module parameter configuration, and business control. Satellite functions are divided into sensing unit, network unit, computing unit, storage unit, execution unit, and BOMS unit. The BOMS unit also includes modules such as attitude and orbit control (AOC), house-keeping system (HKS), satellite tracking, telemetry and remote control (TT&C), and thermal control (TC). **b**, In order to simulate satellite orbit operation, we will mount various modules of iSat on unmanned aerial vehicles, simulate orbital motion, and conduct paper experiments.

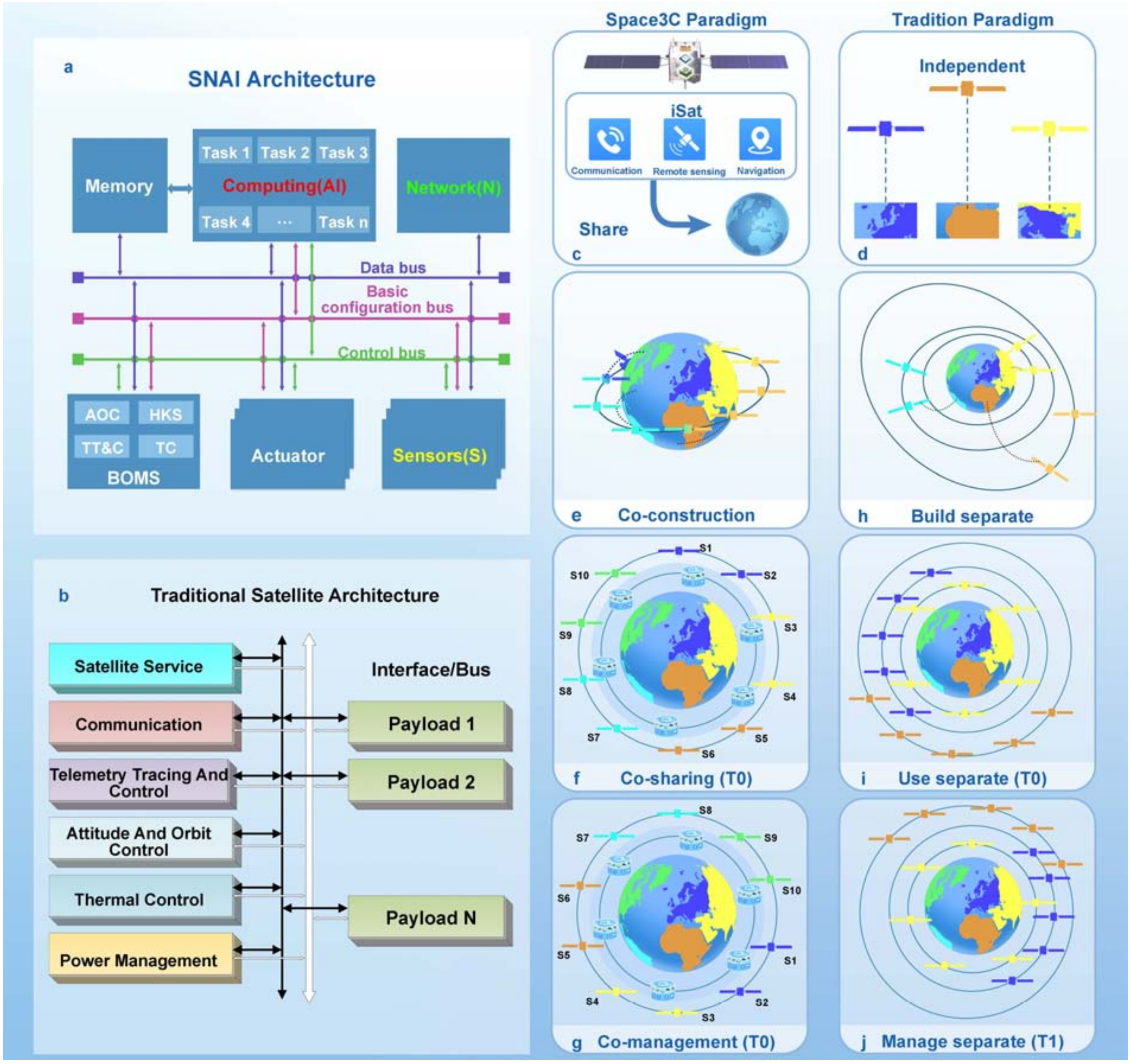

Fig. S2.

ISat architecture and traditional satellite architecture. a. ISat architecture. **b.** Traditonal satellite architecture. **c.** Isat satellite functions can be defined by software according to mission requirements. **d.** Traditional satellite functions are isolated, with one satellite implementing one function to serve a specific area. **e.** Based on the opend and shared paradigm, countries around the world are jointly building a space-based system. **f.** Based on the open and shared paradigm, all satellite resources of space systems are jointly shared by countries around the world. **g.** Based on the opend and shared paradigm, countries around the world jointly maintain and manage space-based systems. **h.** In the traditional model, each country independently builds its own space-based system. **i.** In the traditional model, each country independently uses its own space-based system. **j.** In the traditional model, each country manages its own space-based system.

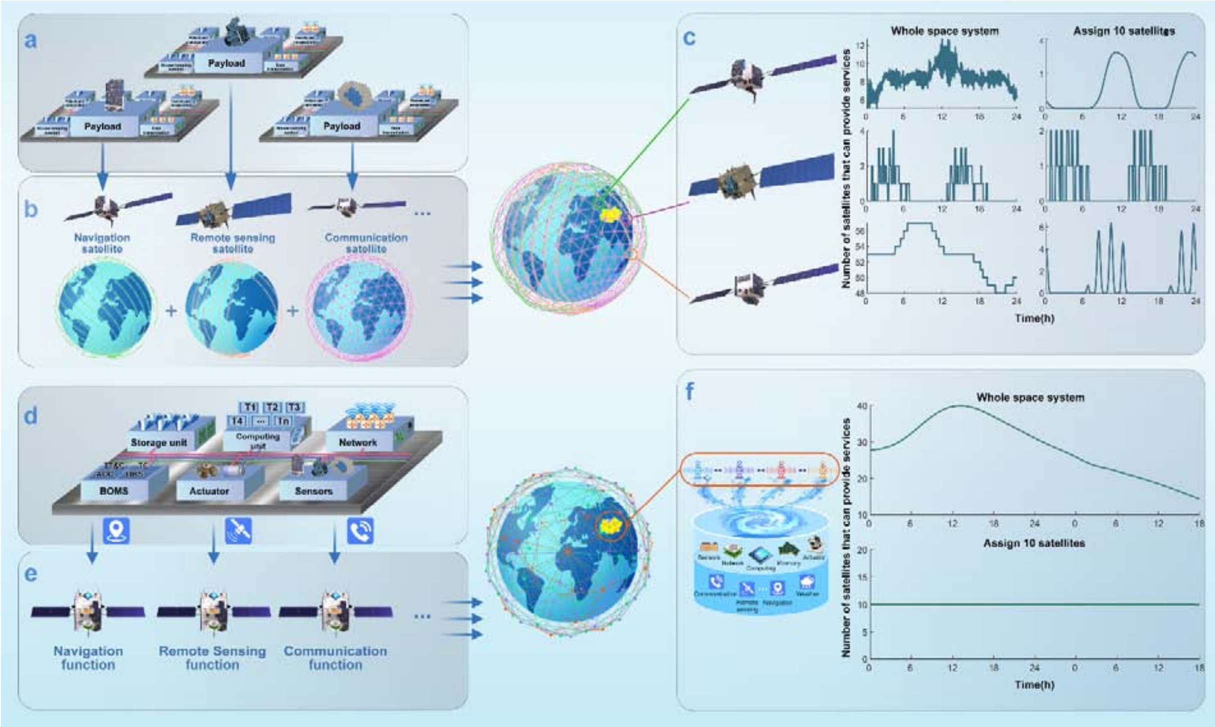

Fig. S3.

The comparison between tradition satellite architecture and SNAI architecture. a. Traditional satellite hardware architecture. **b.** The constellations of three traditional satellites. **c.** The working hours and number of visible satellites provided by three traditional satellite constellations to users in the yellow region, which is a random user aggregation area of three satellite services. **d.** ISat hardware architecture. **e.** The iSat satellite configuration software implements three functions. **f.** The working hours and number of visible satellites provided by the ISS constellation for users in the same region with the scenario in (c).

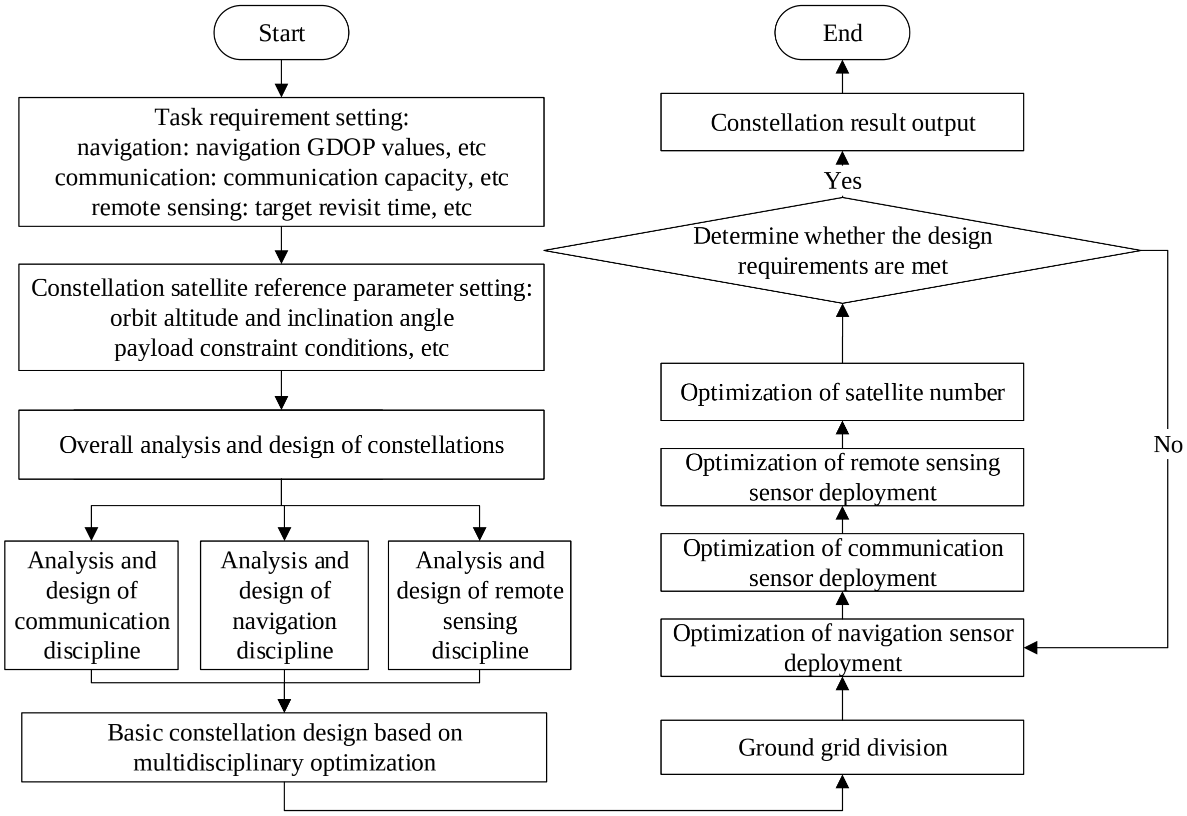

Fig. S4.

Cross-domain fusion constellation design process for ISS. The diagram mainly divides the design process of ISS into two parts. The left part first designs the constellation configuration of ISS, while the right part optimizes the deployment of communication, navigation, and remote sensing sensors, and finally optimizes the number of satellites.

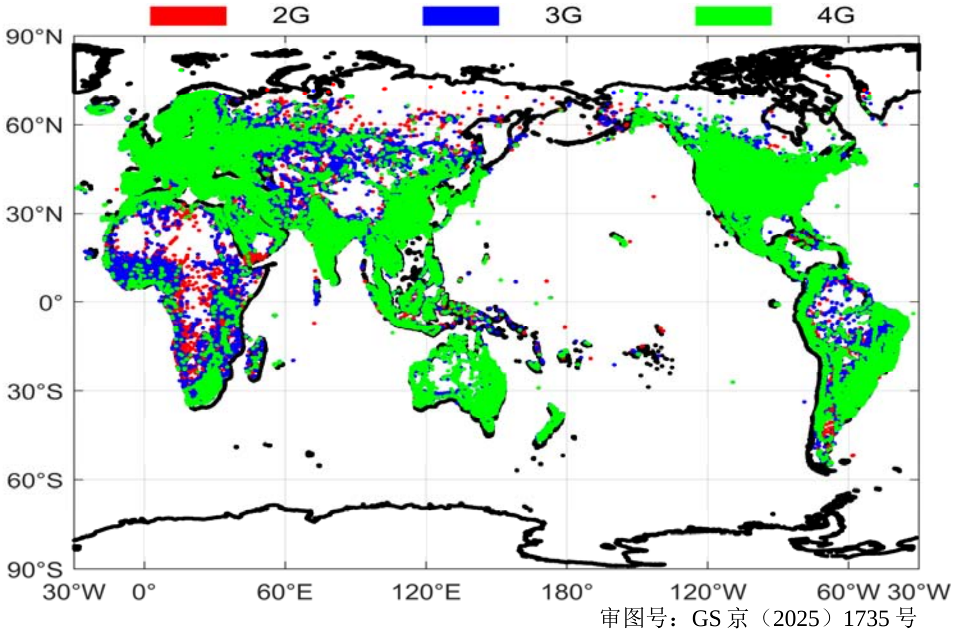

审图号：GS 京（2025）1735 号

Fig. S5.

Communication Coverage Map. The data comes from the global mobile signal development database on the OpenCellID website. Among them, 2G refers to CDMA or GSM systems, 3G refers to UMTS systems, and 4G refers to LTE systems.

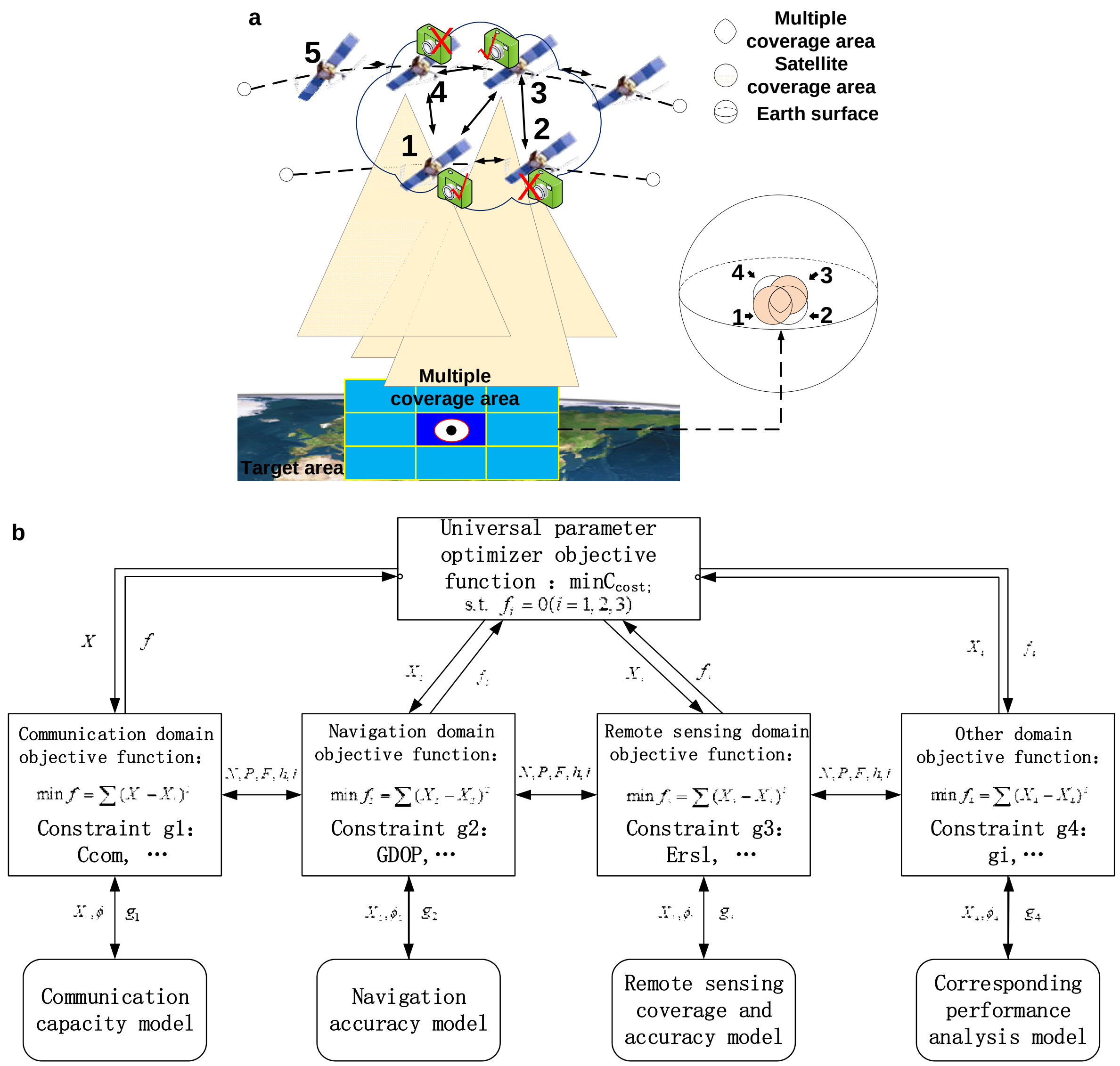

Fig. S6.

Layered collaborative optimization structure diagram for space system design. a. Multiple resource coverage in the target area. **b.** This block diagram has three levels of content from top to bottom, but is divided into two levels in terms of hierarchy. The first layer is the system level optimization objective, the second layer is the subsystem level optimization objective, and the third layer is the subsystem level constraints. From left to right, there are optimization design contents for communication domain, navigation domain, remote sensing domain, and other domains. The other domains indicate that the optimized functional domains are not limited to three domains and can be expanded according to design needs.

a

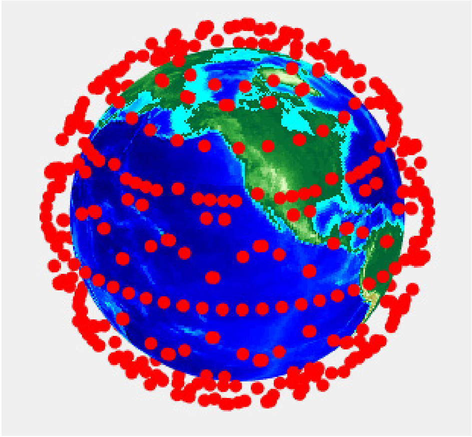

b

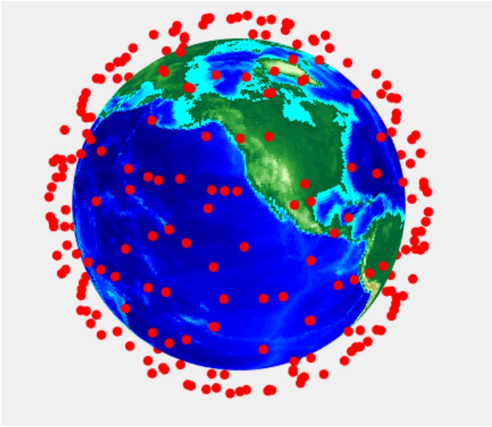

Fig. S7.

Constellations before and after optimization. a, Initial constellation before optimization. **b**, Optimized target constellation.

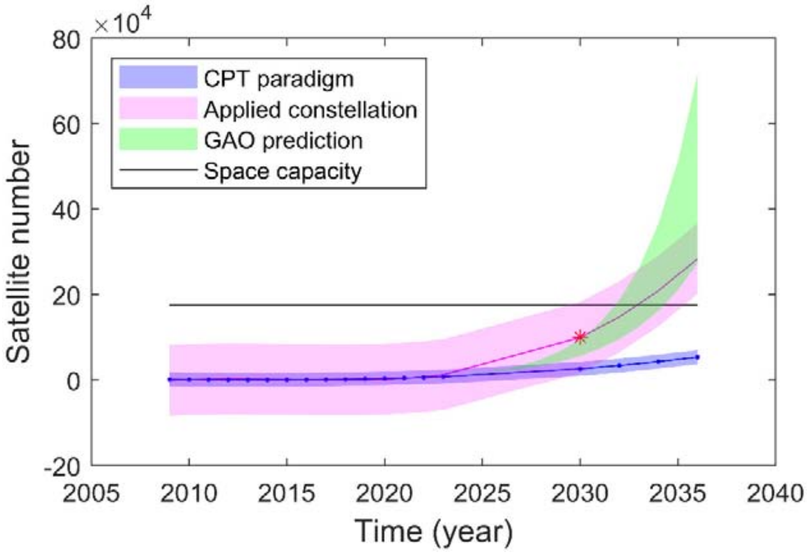

Fig. S8.

Number of in orbit satellites in space orbit. The horizontal axis represents time, and the vertical axis represents the number of satellites. The figure shows the number of satellites in the space environment and the calculated space environment capacity in three scenarios. The red dots represent the intersection of 100000 satellites based on existing constellation planning predictions and GAO predictions. Its prediction is based on the existing constellation application plan, estimated at a growth rate of 30% and 40%.

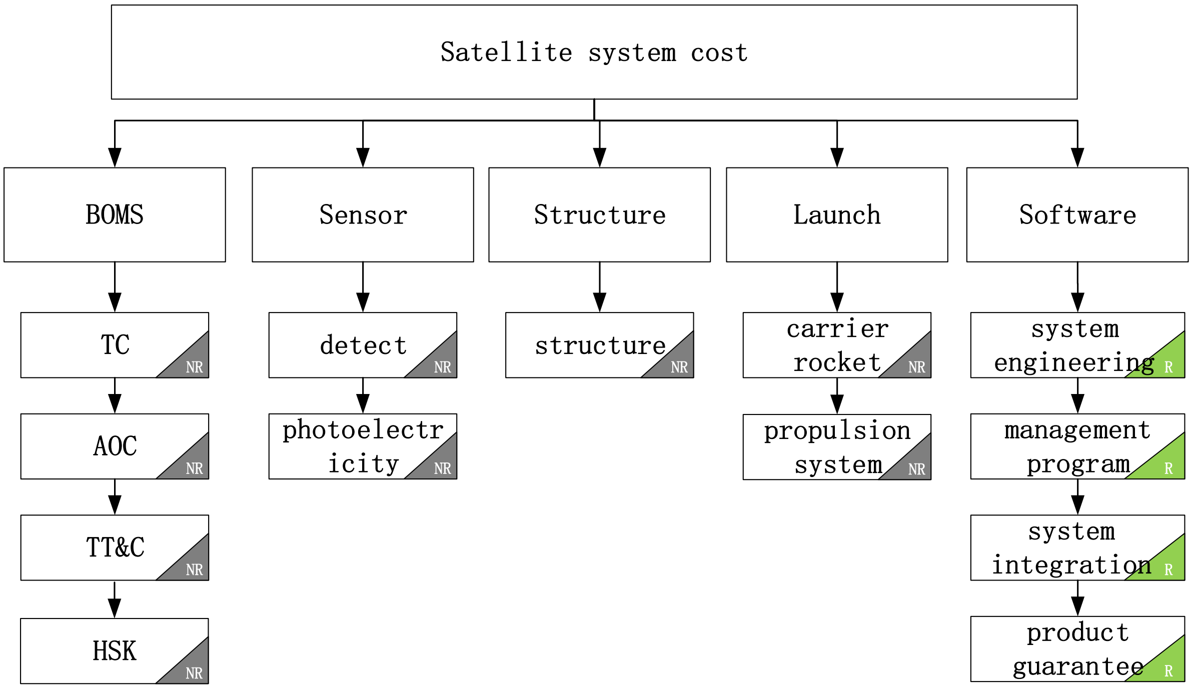

Fig. S9.

Cost estimation model for satellite systems. Split the cost of the satellite system into five modules and focus on the costs below each module. The module with NR in the lower right corner refers to non-recurring costs, while the module with R represents recurring costs.

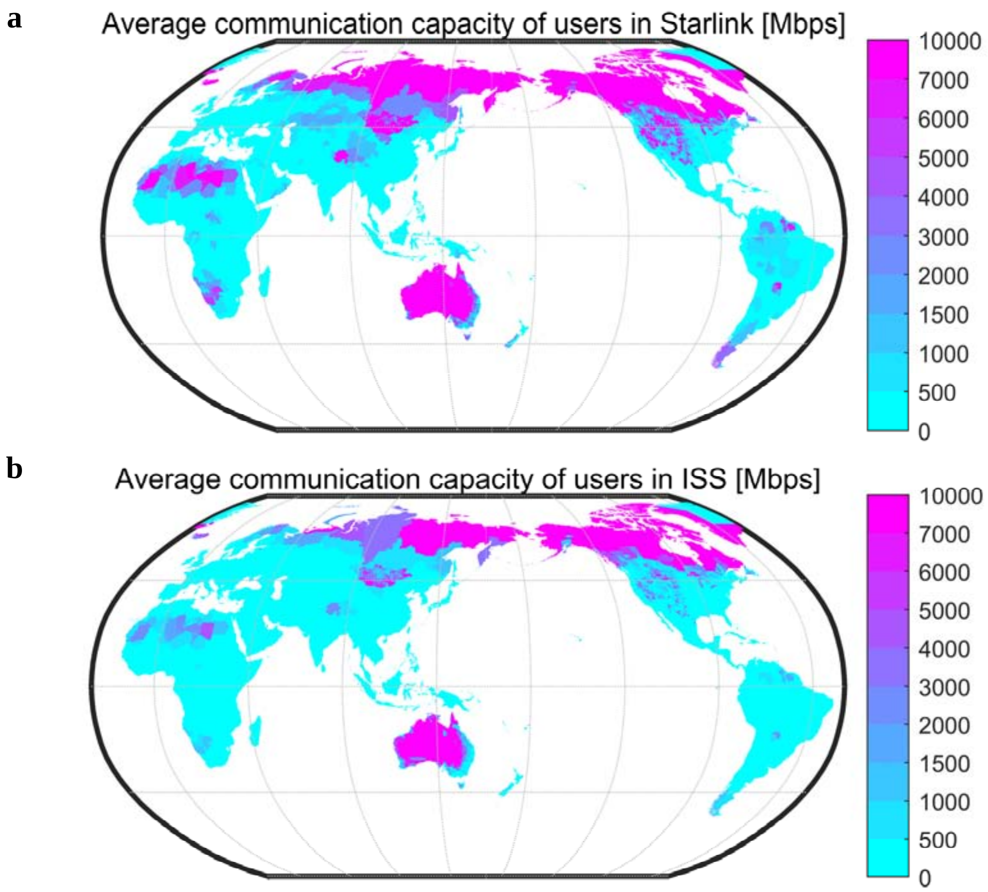

审图号：GS 京（2025）1735 号

Fig. S10.

Heat map of average user communication capacity. a, Average communication capacity of users in Starlink (Mbps). **b**, Average communication capacity of users in ISS (Mbps)..

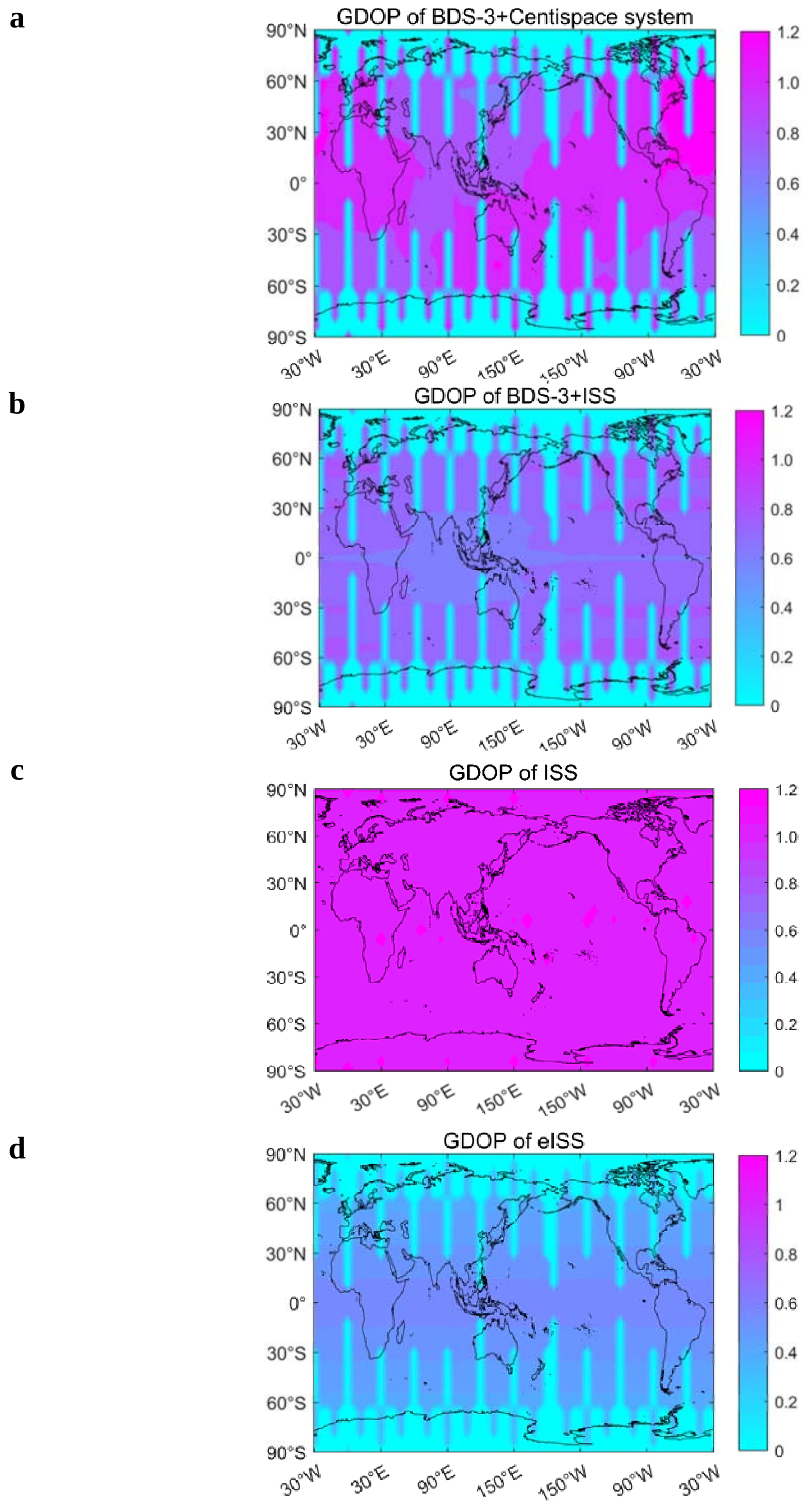

Fig. S11. 审图号：GS 京（2025）1735 号

Heat map of GDOP. a, GDOP of BDS-3 plus Centispace system. **b**, GDOP of BDS-3 plus ISS. **c**, GDOP of ISS.

a

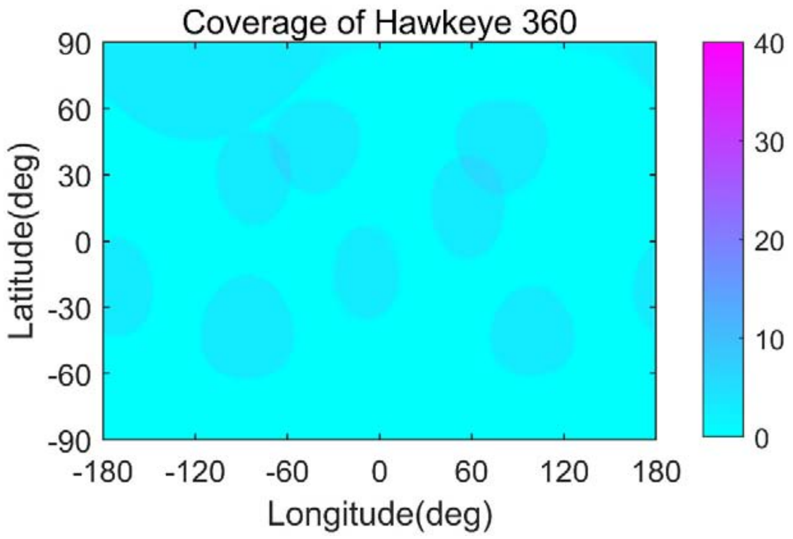

b

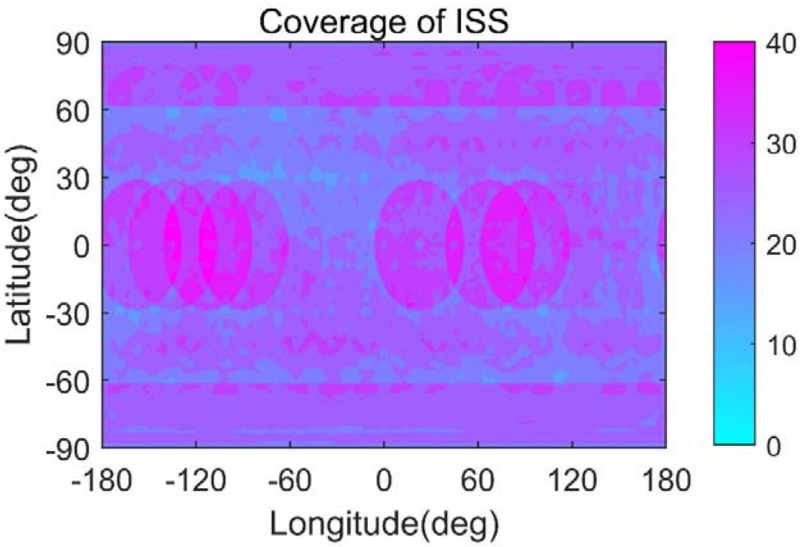

Fig. S12.

Coverage weight for two space systems. a, Hawkeye 360. **b**, Intelligent space system.

a

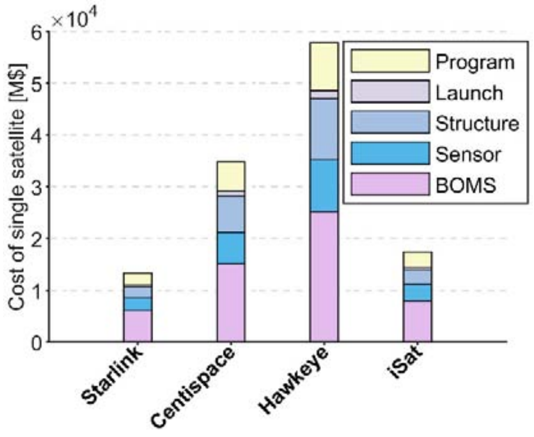

b

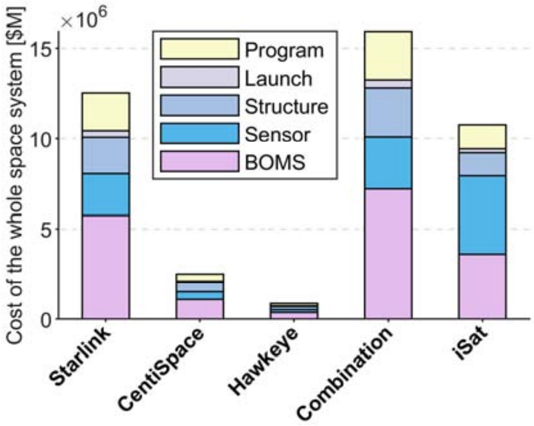

Fig. S13.

Cost of single satellite and the whole space system. a, Cost of single satellite. **b**, Cost of the whole space system.

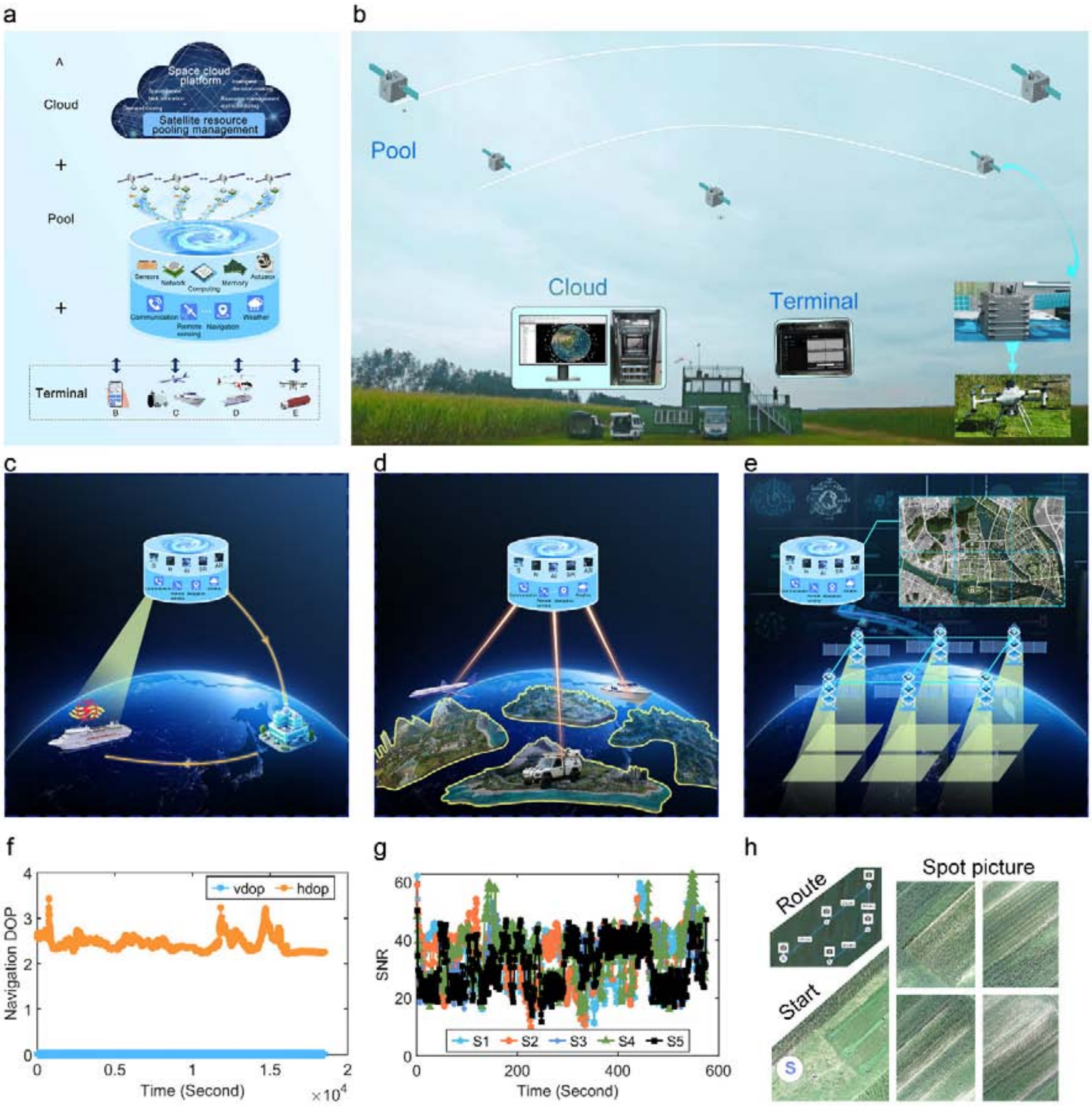

Fig. S14.

ISS architecture based on 'cloud-pool-terminal'. a, CPT architecture diagram. **b,** Experiment scenario. **c,** Satellite emergency communication scenarios under international search and rescue. **d,** Navigation enhanced scenarios. **e,** Collaborate with multiple satellites to capture an area and synthesize a large-scale satellite image. **f,** Navigation signal in navigation enhanced scenarios. **g,** Results of satellite emergency communication scenarios under international search and rescue. **h,** Results of collaborate with multiple satellites to capture an area and synthesize a large-scale satellite image.

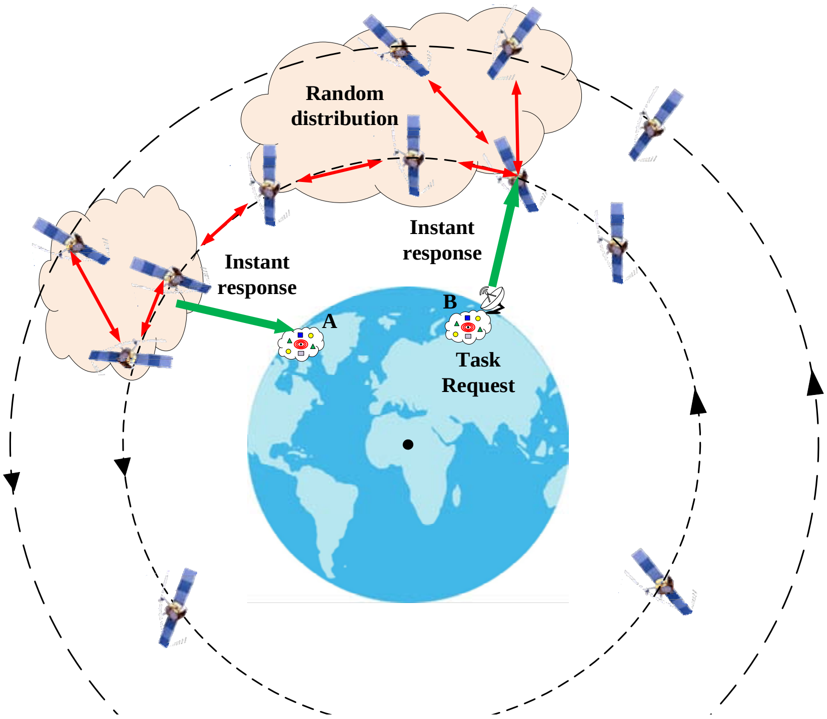

Fig. S15.

Simulation task scenario. This scenario is for ground users to request tasks from space-based systems, and space-based systems respond to services. Ground users can submit task requirements to space-based systems through handheld terminals (Area A) or ground base stations (Area B). The satellites connected to the user share resources throughout the entire constellation through a constellation, collaborate to complete tasks proposed by the user, and provide timely and fast services. From the perspective of the entire system, users do not need to know the satellite's orbit and other information, and it is a service mode of random allocation and instant response.

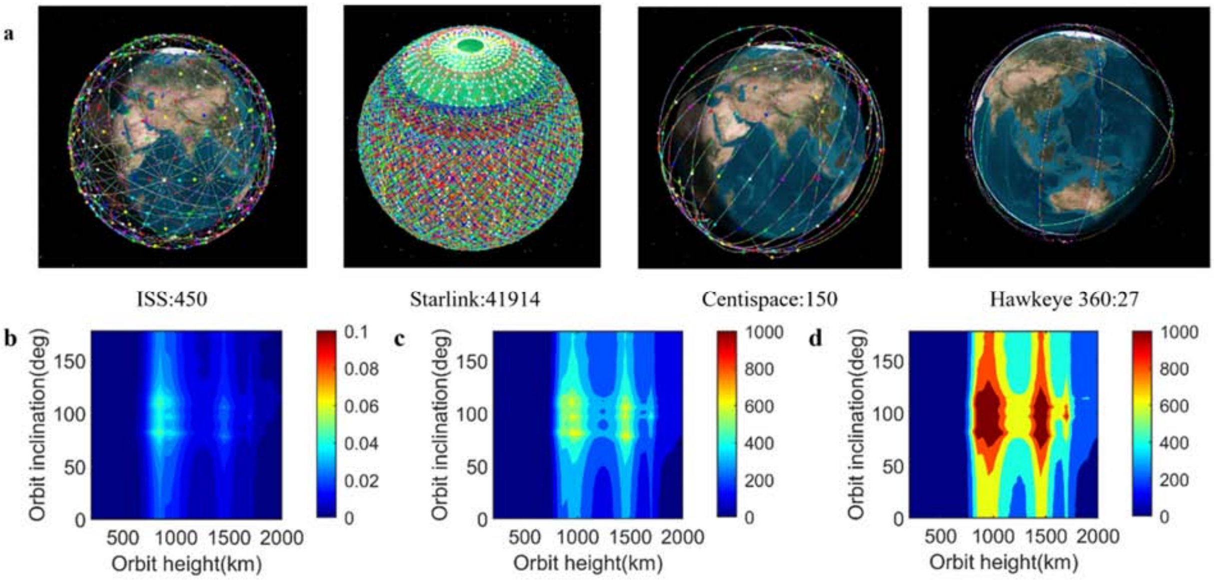

Fig. S16.

Schematic diagram of each space system and OIS Based on space debris cumulative cross sectional area grid. a, Four space systems and the satellite number of each. **b,** OIS of space orbit resources before 2021. **c,** OIS after deploying ISS. **d,** OIS after deploying combination system.

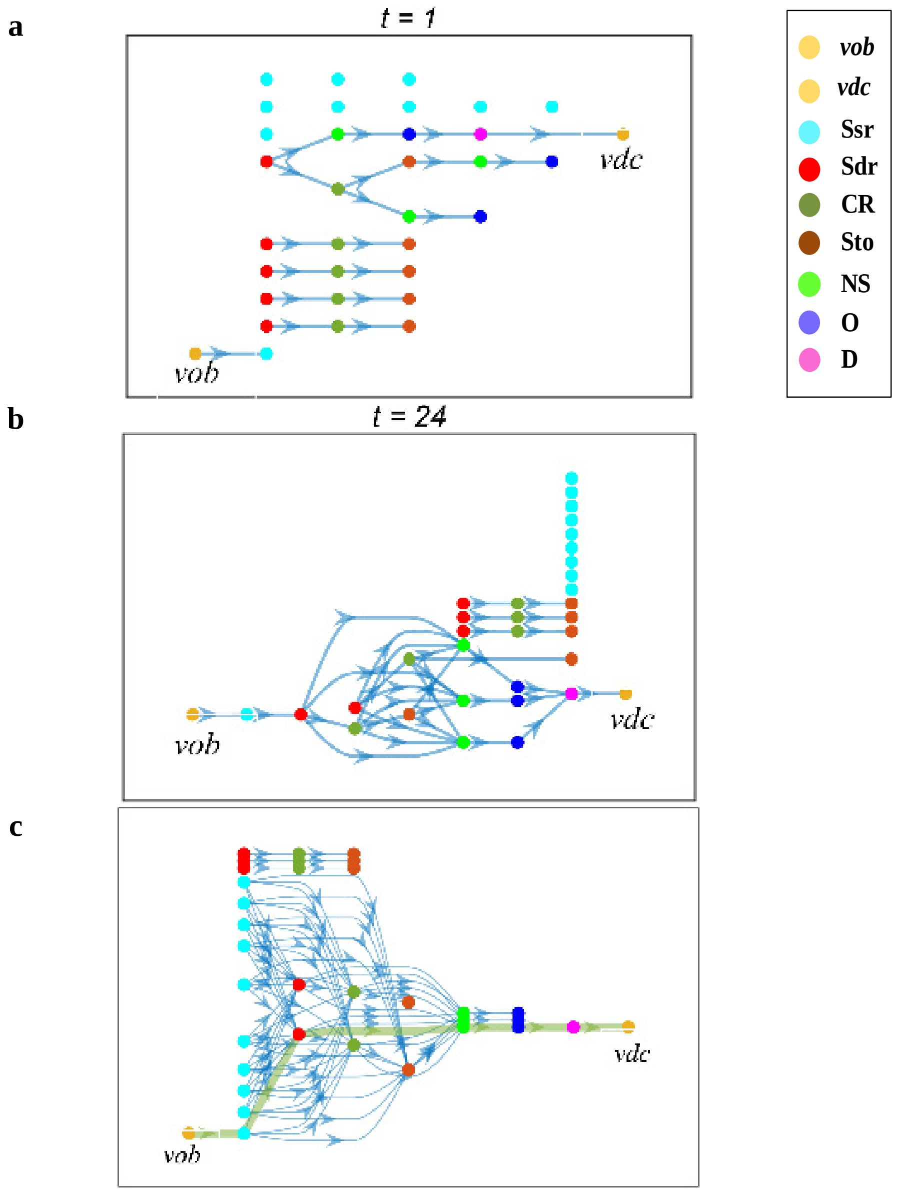

Fig. S17.

Time variant diagram of resource expansion. a, Time variant diagram of resource expansion in traditional space systems at T=1. **b**, Time variant diagram of resource expansion in traditional space systems at T=24. **c**, Time variant diagram of resource expansion in ISS. The reason why the **c** is not marked with time is that under the CPT paradigm based on ISS, the resource sequence diagram at each time step is similar to this diagram, with more resource paths to complete tasks.

a

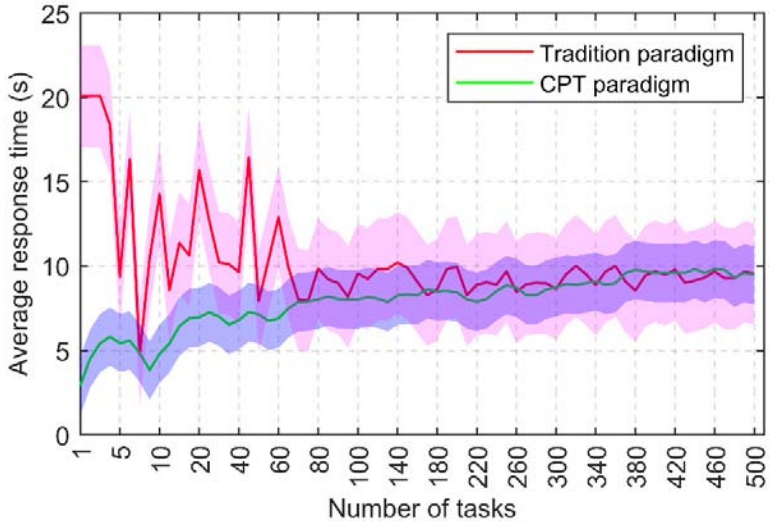

b

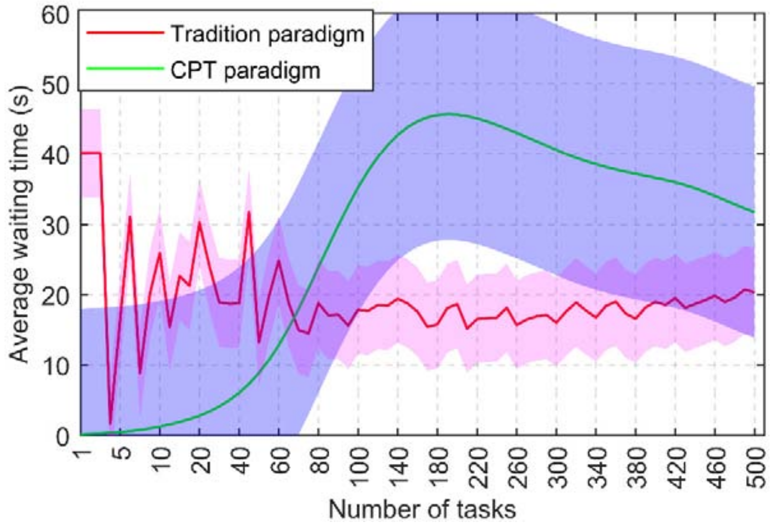

Fig. S18.
a, the average response time of a task. **b,** The average waiting time for a task.

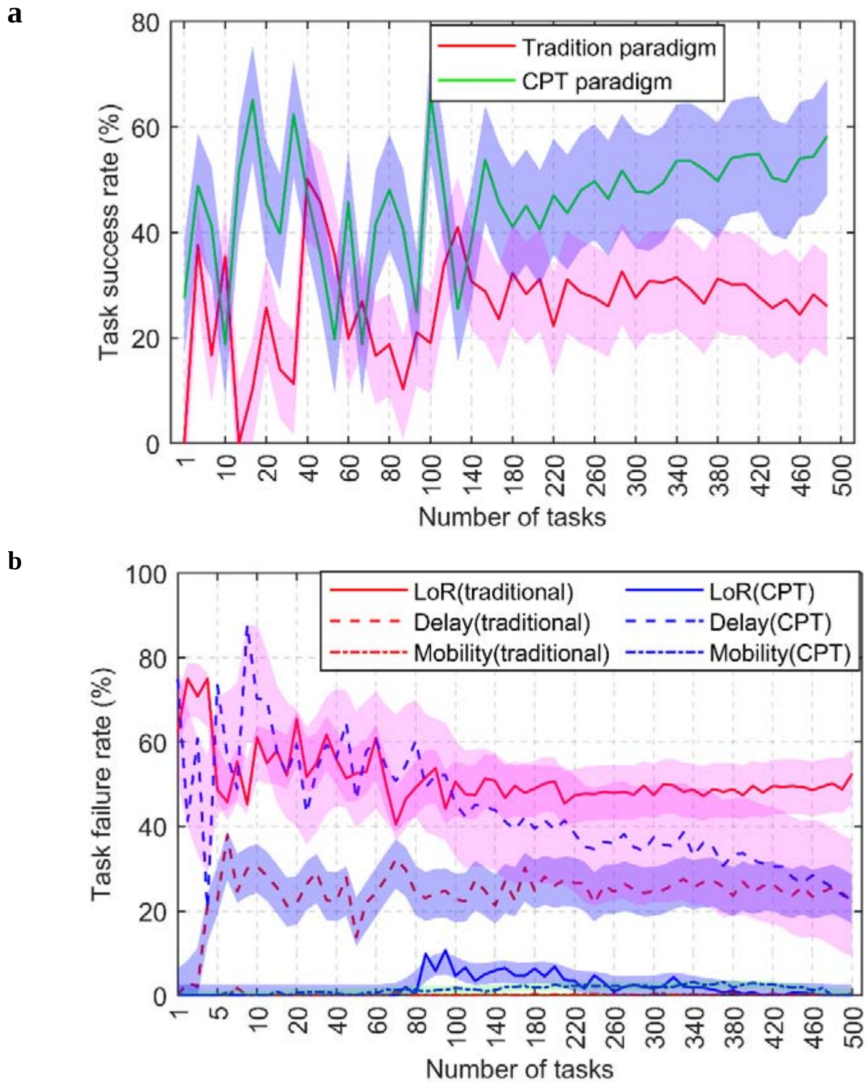

Fig. S19.

a, Task success rate. **b,** Task failure rate.

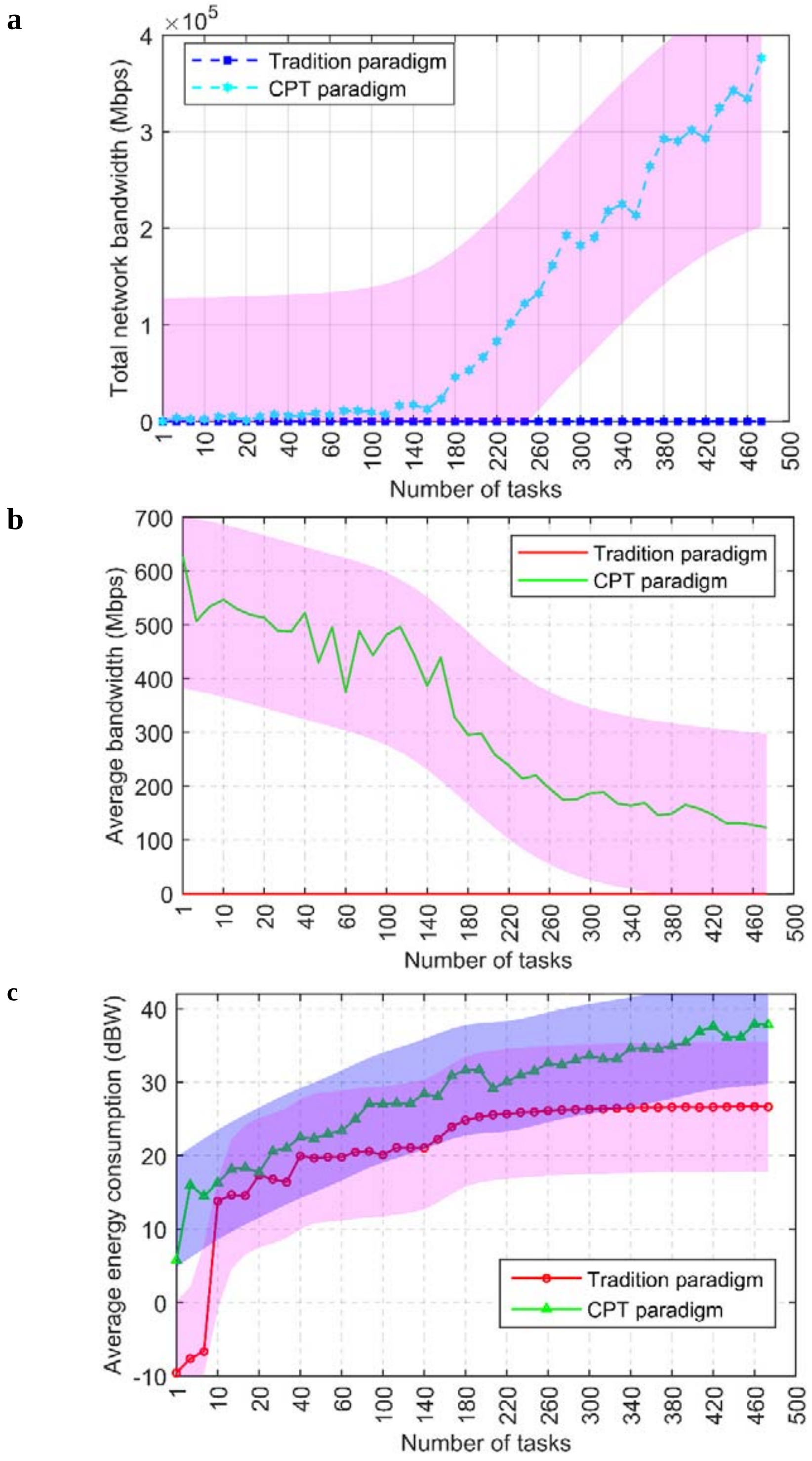

Fig. S20.

a, Totalbandwidth usage for each task. **b,** Average bandwidth for each task. **c,** Average energy consumption per satellite.

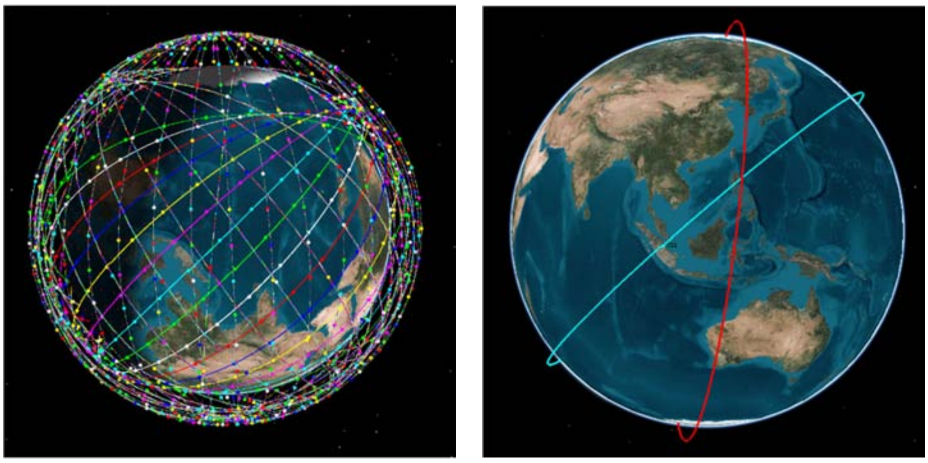

a b

Fig. S21.

a, The number of ISS's satellites has expanded to 1320. **b,** Two typical space users.

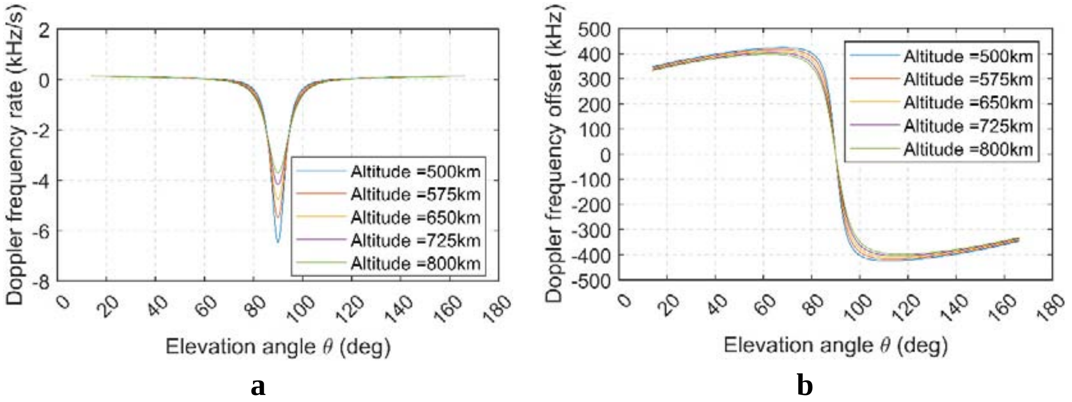

Fig. S22.

Doppler frequency rate and offset for ISS constellation. a, Doppler frequency rate. **b,** Doppler frequency offset.

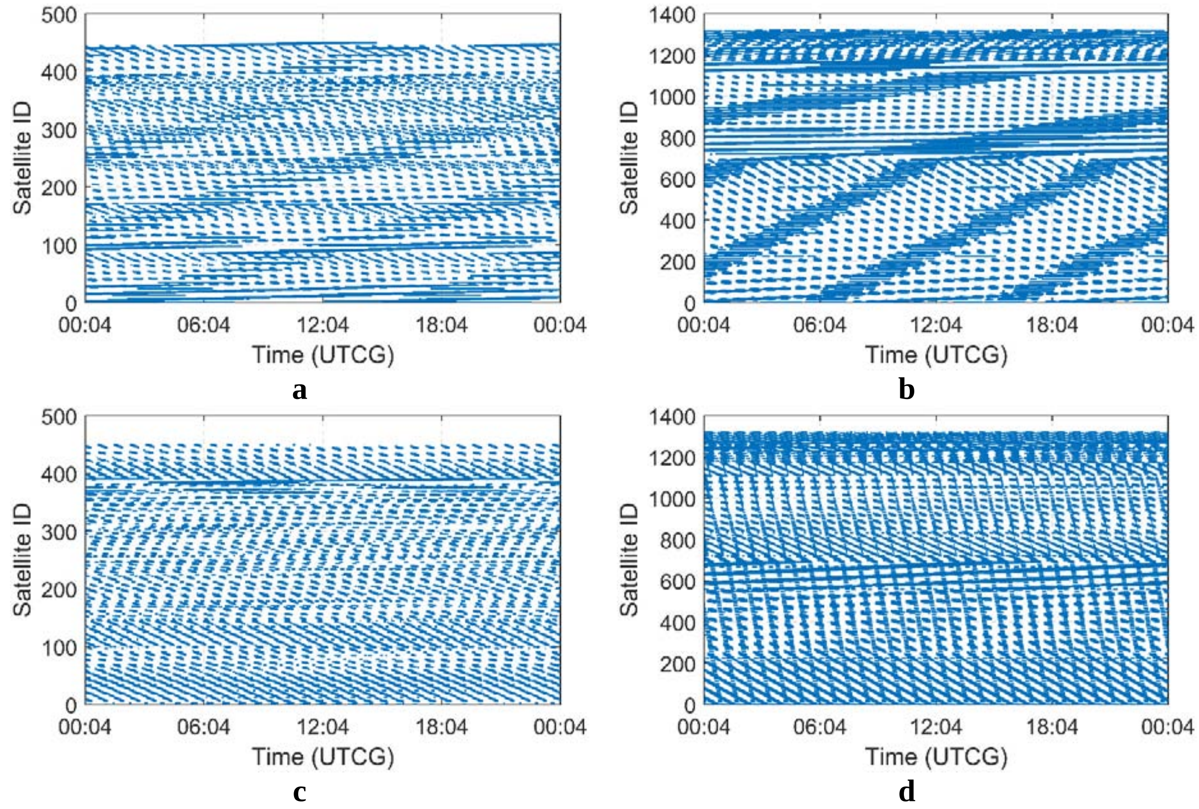

Fig. S23.

The availability satellite for CSS or EOSS during simulation time. a, CSS with ISS(450). **b,** CSS with ISS(1320). **c,** EOSS with ISS(450). **d,** EOSS with ISS(1320).

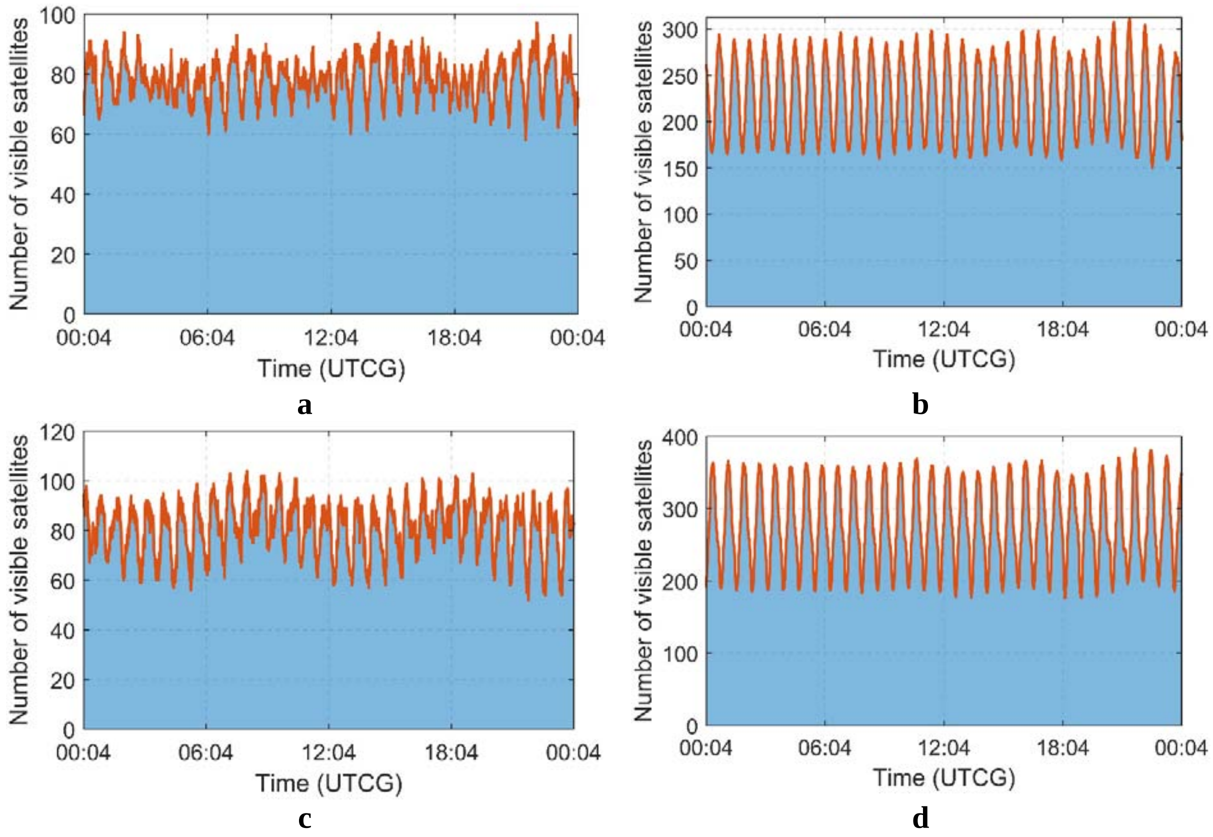

Fig. S24.

The availability satellite number for CSS or EOSS during simulation time. a, CSS with ISS(450). **b,** CSS with ISS(1320). **c,** EOSS with ISS(450). **d,** EOSS with ISS(1320).

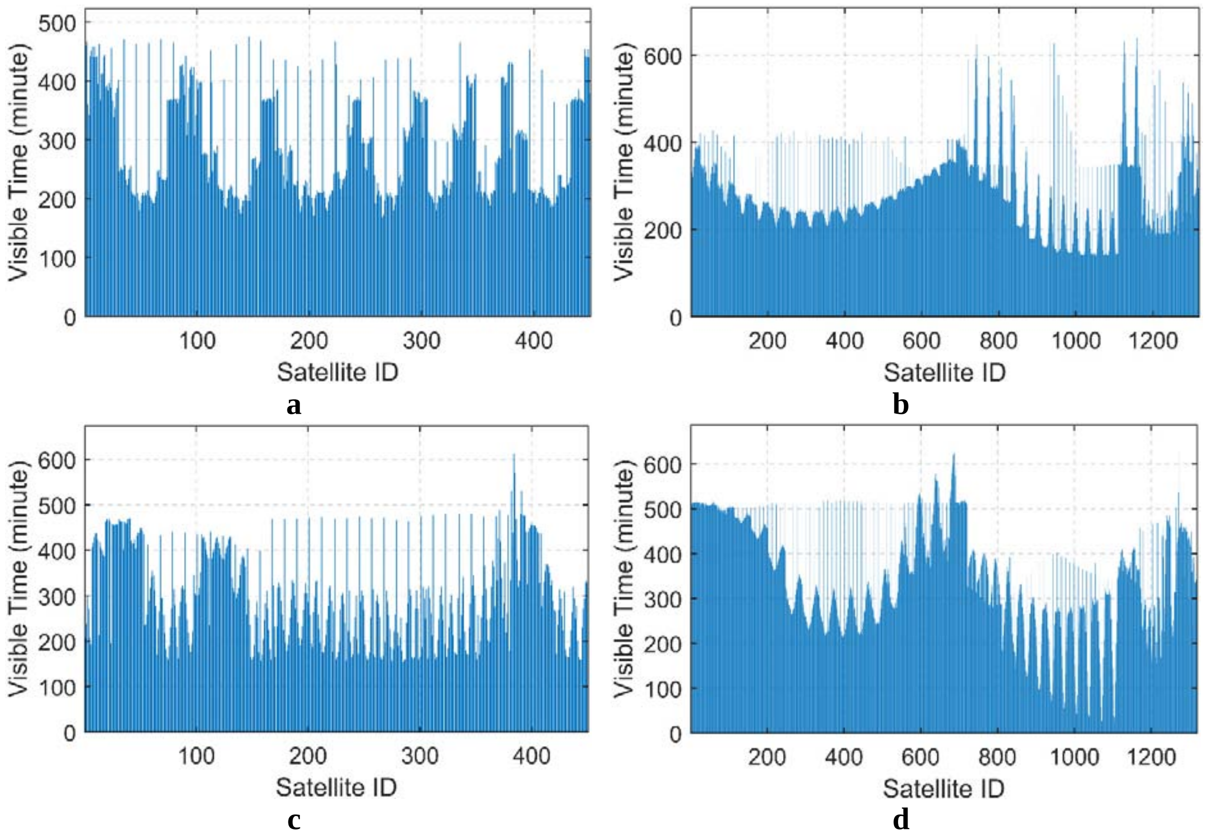

Fig. S25.

The availability duration of each satellite for CSS or EOSS during simulation time. a, CSS with ISS(450). **b,** CSS with ISS(1320). **c,** EOSS with ISS(450). **d,** EOSS with ISS(1320).

a

b

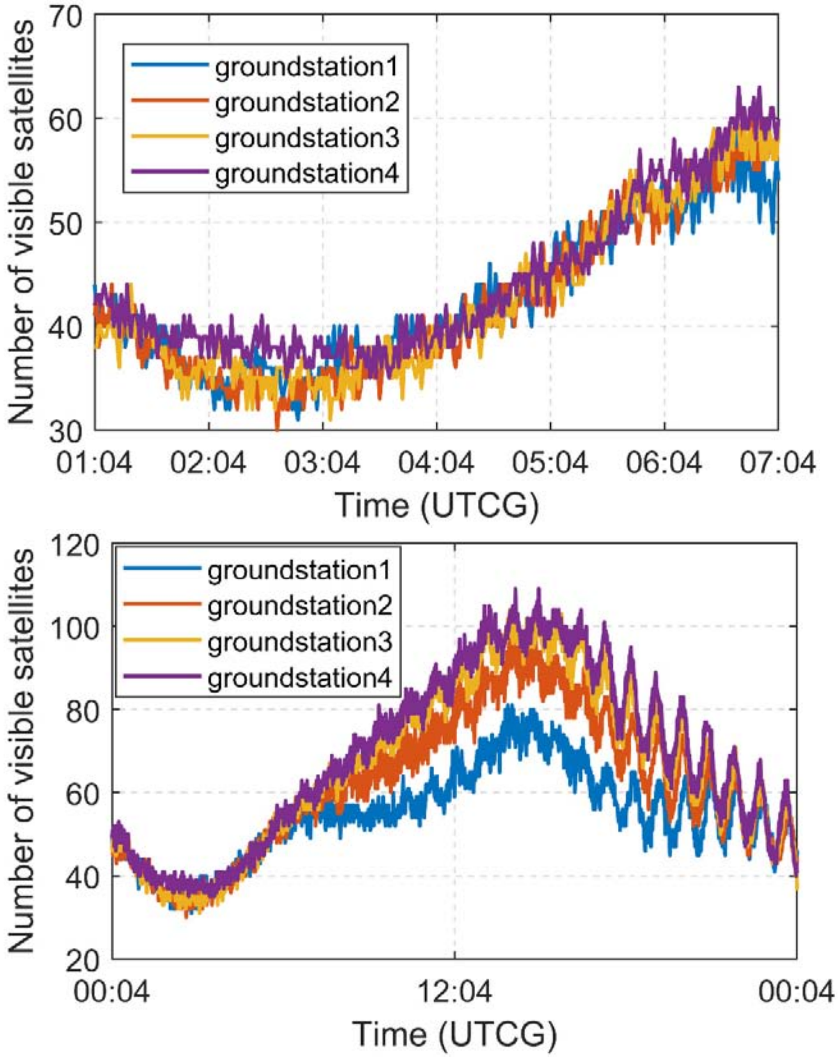

Fig. S26.

Number of visible satellites in the view of four ground stations. a, Simulation time: 6 hours. **b,** Simulation time: 24 hours.

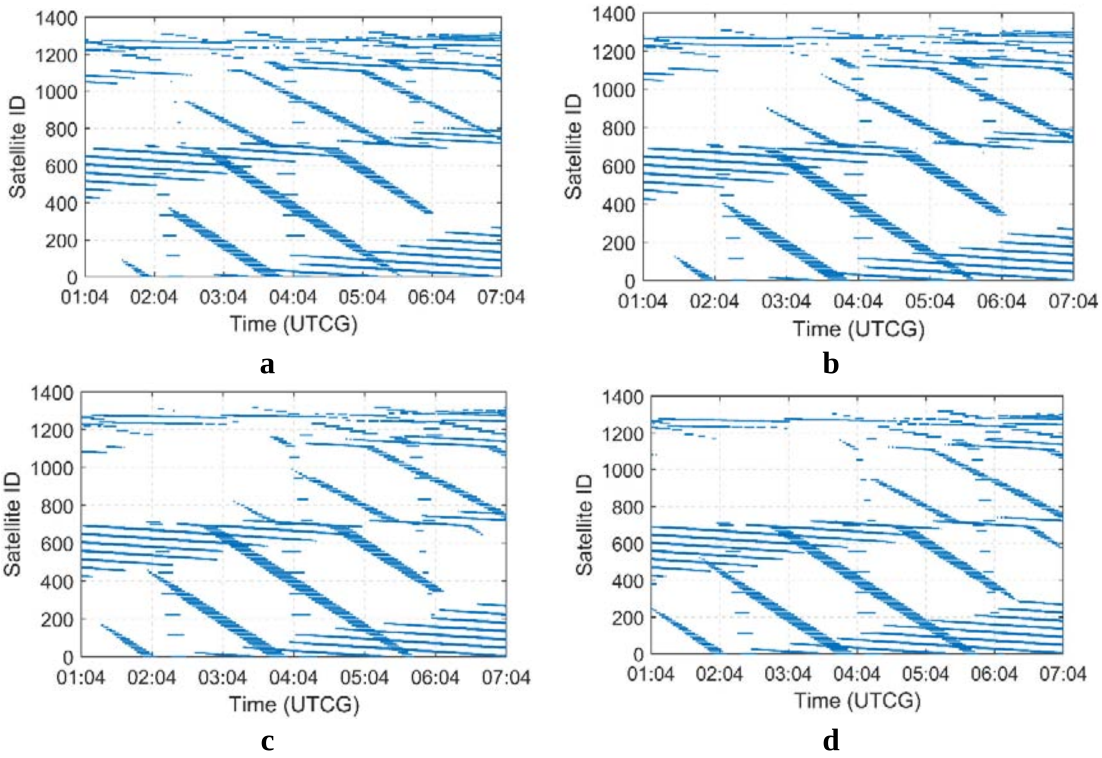

Fig. S27.

Visibility of ground stations by various satellites during simulation time (6 hours). a, Groundstation1. **b,** Groundstation2. **c,** Groundstation3. **d,** Groundstation4.

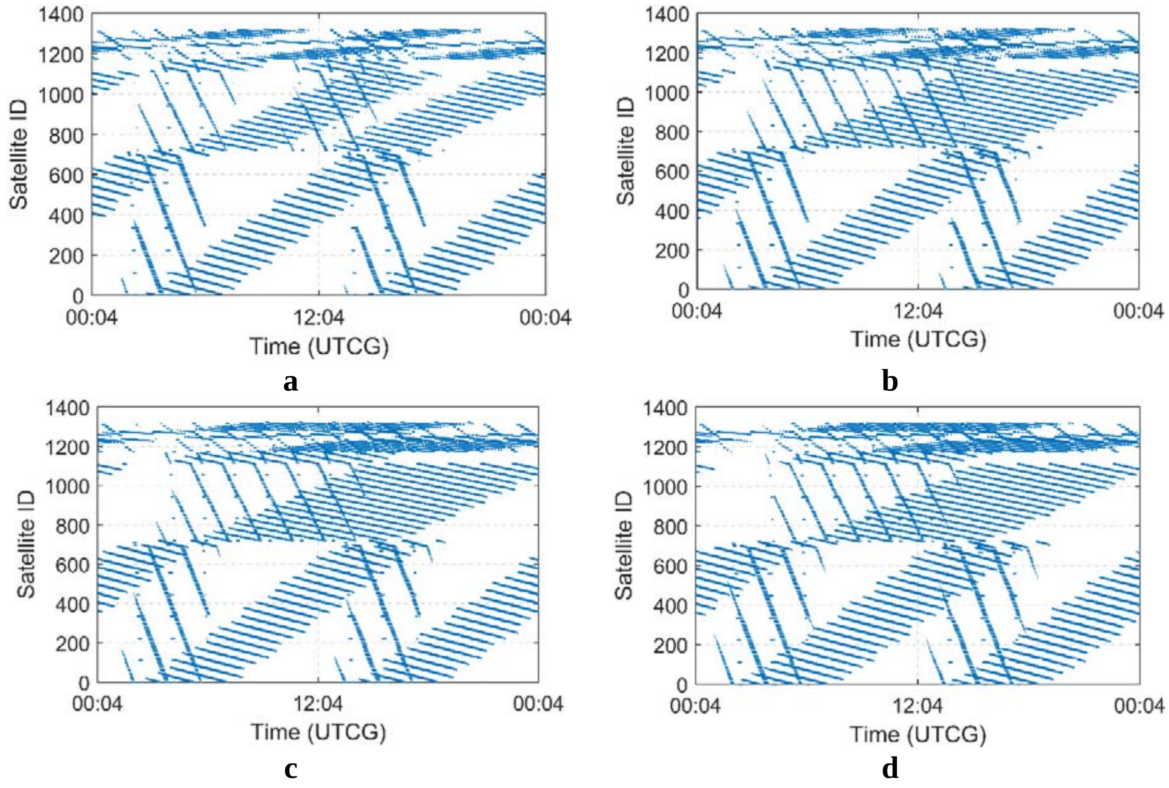

Fig. S28.

Visibility of ground stations by various satellites during simulation time (24 hours). a, Groundstation1. **b,** Groundstation2. **c,** Groundstation3. **d,** Groundstation4.

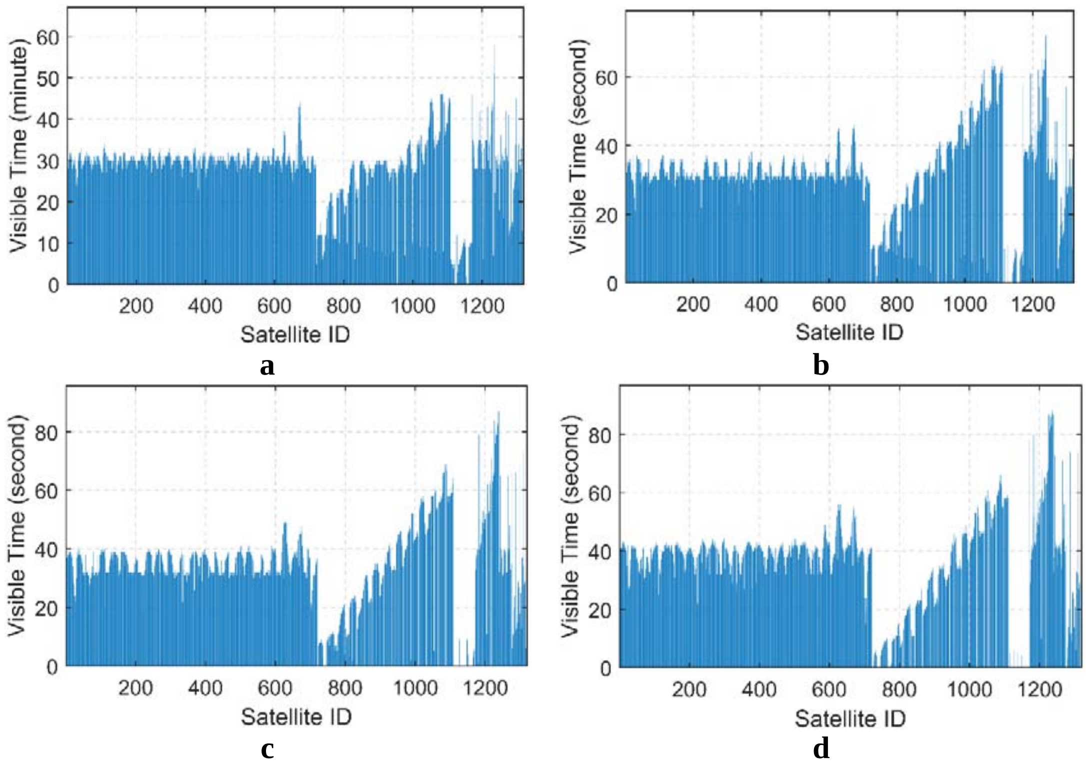

Fig. S29.

The visible duration of each satellite to the ground station during simulation time (6 hours). **a,** Groundstation1. **b,** Groundstation2. **c,** Groundstation3. **d,** Groundstation4.

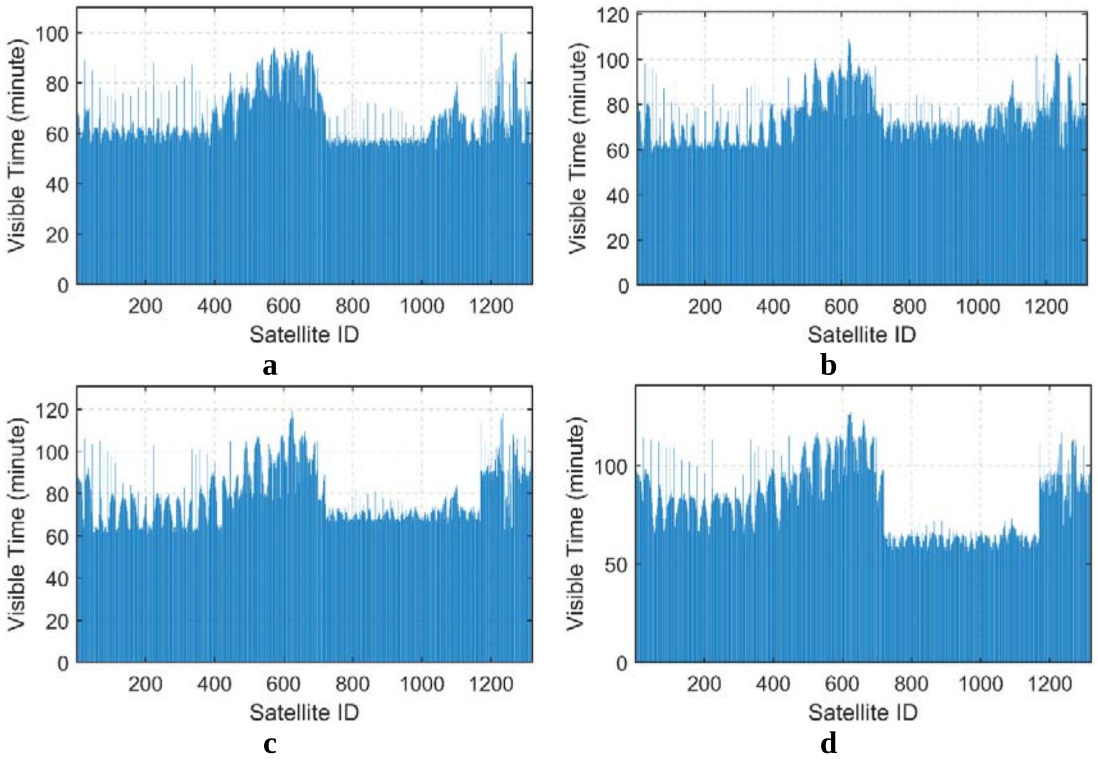

Fig. S30.

The visible duration of each satellite to the ground station during simulation time (24 hours). a, Groundstation1. **b,** Groundstation2. **c,** Groundstation3. **d,** Groundstation4.

Table S1:Communication model parameter value table.

Model parameter	Value	Unit
Path loss index	2	/
Transmission power of each user terminal	2	W
Constant power gain factor	43.3	dBi
Minimum elevation angle θ_{min} of user terminal	10~18	deg
The number of subchannels	1000	/
End user density	4×10^{-6}	km^{-2}
Return demand for each user terminal	60~160	Mbps
Communication bandwidth B in the Ka frequency band	800	MHz
Noise density in the Ka frequency band	-203	dBm/Hz

Table S2: Number of satellite launches from 2008 to 2023.

Time/year	2009	2010	2011	2012	2013
Satellite number	61	66	80	96	118
Time/year	2014	2015	2016	2017	2018
Satellite number	135	111	240	321	299
Time/year	2019	2020	2021	2022	2023
Satellite number	1122	1673	1747	2118	2891

Table S3: Number of satellites in orbit from 2008 to 2023.

Time/year	2009	2010	2011	2012	2013
Satellite number	986	997	1033	1091	1187
Time/year	2014	2015	2016	2017	2018
Satellite number	1262	1364	1462	1778	2027
Time/year	2019	2020	2021	2022	2023
Satellite number	2272	3256	4800	6905	9029

Table S4: Satellite cost model parameter table.

Model		SSCM recurring + nonrecurring
Structure	ST	$407+19.3m_1\log_{10}(m_1)+355+5.7m_2^2$
ADCS	ADCS	$1850+11.7m_3^2$
EPS	EPS	$1261+539m_4^{0.72}$
Telemetry	TT&C	$486+55.5m_5^{1.25}$
Bus cost	SC_b	ADCS+EPS+TT&X+ST
Payload	PL	$0.4{*}SC_b$
Satellite cost	SC	SC_b+PL
LA&T	LA&T	$0.139*SC_b$
Program	P	$0.229*SC_b$
Aerospace and ground	AGE	$0.066*SC_b$
Launch Ops	LOPS	$0.061*SC_b$

Table S5: System impact parameters on spatial environment.

Name	n_{sat}	α-R_E [km]	i [°]	m [kg]	A [m^2]	Density	SVCP	OIS
Starlink	720	1200	87.9	187200	1944	1.1337e-10	2.6412e-6	365.35
Hawkeye	15	575	97-98	225	225	1.2000e-13	2.6290e-6	0.03
Centispace	150	975/1200	55/85	97	249	5.4679e-12	2.6411e-6	2.87
combination	807	/	/	249345	2418	1.1854e-10	6.9122e-6	397.87
ISS	450	900	/	126000	1350	9.7200e-11	4.9281e-6	186.42

Table S6: The Relationship between Power and Number of Satellites in Collaborative Enhancement.

Target power	-90dBm	-100dBm	-110dBm	-120dBm
Required transmission power	55.60KW	5.56KW	556W	55W
Number of satellites required	36	12	4	2
Synthetic efficiency	85.8%	77.2%	69.5%	27.8%
Time synchronization accuracy	24.6ps	31.7ps	37.2ps	64.6ps

Table S7: Two Global GDOP Values for Low Earth Orbit Constellations to Improve GDOP.

System solution	Global GDOP			GDOP improvement（%）		
	Mean	Maximum	Minimum	Mean	Maximum	Minimum
BDS	1.5486	1.8552	1.2214	-	-	-
BDS+Centispace	1.0472	1.4651	0.7673	32.02	50.70	11.02
BDS+ISS	0.7524	0.9140	0.6072	51.07	61.30	35.13

Table S8. Constellation Parameters Used in Task Simulation.

Name	ISS	eISS	Starlink	Centispace	Hawkeye 360	Combination
n_{sat}	450	1320	42000(720)	150	15	885
α-R_E [km]	900/800	1200/1000 /800	1130/1275	975/1200	575	/
i [°]	/	/	74/81	55/85	97-98	/

Table S9. Application Resource Configuration Table.

	Navigation	Remote sensing	Communication
Max_delay/s	5	5	200
Container_size/KB	2500	9000	13000
Task_length/MI	60000	15000	200000
Required_core/PE	3	2	2
Required_memory/MB	100	500	300
Required_sensor	SDR	camera	(wave)
Poission_interaarrival/s	40	10	30
Target	No target area	Targeted area	Visible to another star

Table S10. The latitude and longitude of the ground station.

ID	1	2	3	4
Latitude(°)	22	29	36	43
Longitude (°)	112	112	112	112

References

1. Mao Y, Wu Y, Ju X, et al. Dynamic Management Topology Construction, Evolution, and Maintenance of Low Earth Orbit Mega-Constellation. Space Sci Technol. 2025, 5:0248. DOI: 10.34133/space.0248.
2. Valenzuela AQ, Reinke K, Jones SD. A New Methodology to Assess Spatial Response Models for Satellite Imagers using the Optical Design Parameters of a Generic Sensor as Independent Variables. IEEE Transactions on Geoscience and Remote Sensing, 2023, 61: 1. DOI: 10.1109/TGRS.2023.3270433.
3. Wang YX. Technologies for Space-based Network Intelligent Satellite Mission Collaboration. Ph.D. thesis, National University of Defense Technology, 2020.
4. Yang Y, Mao Y, Ren X, et al. Demand and key technology for a LEO constellation as augmentation of satellite navigation systems. Satellite Navigation 5, 11 (2024). DOI: 10.1186/s43020-024-00133-w.
5. Xu XY, Wang CH, Jin ZH. Design, analysis and optimization of random access inter-satellite ranging system. Journal of Systems Engineering and Electronics, 2020, 31(5): 871-883. DOI: 10.23919/JSEE.2020.000067.
6. Ritchie H, Rodés-Guirao L, MathieuE, et al. (2023) - "Population Growth". Published online at OurWorldInData.org. Retrieved from: 'https://ourworldindata.org/population-growth'.
7. Zhu Y, Liu R, Sheng M, et al. Utilization and Analysis of Resource Mobility in Space Information Networks. Journal of Communications and Information Networks, 2019, 4(1): 67-77. DOI: 10.23919/JCIN.2019.8916647.
8. Sheng M, Zhou D, Bai W, et al. Coverage enhancement for 6G satellite-terrestrial integrated networks: performance metrics, constellation configuration and resource allocation. Science China Earth Sciences, 2023,66(3). DOI: 10.1007/s11432-022-3636-1.
9. Hao Q, Sheng M, Zhou D, et al. A Multi-Aspect Expanded Hypergraph Enabled Cross-Domain Resource Management in Satellite Networks. IEEE Transactions on Communications, 70(7), pp. 4687-4701, July 2022. DOI: 10.1109/TCOMM.2022.3174886.
10. https://www.opencellid.org/.
11. Del Portillo Barrios I. Space and aerial architectures to expand global connectivity. Massachusetts Institute of Technology, Department of Aeronautics and Astronautics, 2020.
12. Hu J, Huang H, Yang L, et al. A multi-objective optimization framework of constellation design for emergency observation. Advances in Space Research, 2021, 67(1): 531-545. DOI: 10.1016/j.asr.2020.09.031.
13. Gu Q, Xu Q, Li X. An improved NSGA-III algorithm based on distance dominance relation for many-objective optimization. Expert Systems with Applications, 2022, 207: 117738. DOI: 10.1016/j.eswa.2022.117738.
14. He BY, Cao J, Zhou QR, et al. Multi objective optimization design of mixed constellation for ground cooperative observation. Journal of Northwestern Polytechnical University, 2021, 39 (1): 224-232. DOI: 10.1051/jnwpu/20213910224.
15. Deng R, Di B, Zhang H, et al. Ultra-Dense LEO Satellite Constellations: How Many LEO Satellites Do We Need?. IEEE Transactions on Wireless Communications, 20, no. 8, pp. 4843-4857, Aug. 2021. DOI: 10.1109/TWC.2021.3062658.
16. Yang M, Dong X, Hu M. Design and Simulation for Hybrid LEO Communication and Navigation Constellation. 2016 IEEE Chinese Guidance, Navigation and Control Conference (IEEE CGNCC2016), 2016. DOI: 10.1109/CGNCC.2016.7829041.
17. Zhang Y, Li Z, Li R, et al. Orbital design of LEO navigation constellations and assessment of

their augmentation to BDS. Advances in Space Research, 2020, 66(8):1911-1923. DOI: 10.1016/j.asr.2020.07.021.
18. Chen X, Dai G, Reinelt G, et al. A Semi-Analytical Method for Periodic Earth Coverage Satellites Optimization. IEEE Communications Letters, 22, no. 3, pp. 534-537, March 2018. DOI: 10.1109/lcomm.2017.2780107.
19. Meng B. Multi objective and multidisciplinary optimization design of constellations and full life flight mission planning. Beijing University of Aeronautics and Astronautics, doctoral thesis two thousand and nine. 2009.
20. Wang XR, Xing GL, Zhang YF, et al. Integrated coverage and connectivity configuration in wireless sensor networks. Proc. 1st Int. Conf. Embedded Netw. Sensor Syst. (SenSys), 2003,pp. 28–39. DOI: 10.1145/958491.958496.
21. Zhu Y, Bai W, Sheng M, et al. Joint UAV Access and GEO Satellite Backhaul in IoRT Networks: Performance Analysis and Optimization. IEEE INTERNET OF THINGS JOURNAL, 2021, 8(9): 7126-7139. DOI: 10.1109/JIOT.2020.3038691.
22. Muttiah R. Satellite constellation design for 5G wireless networks of mobile communications. International Journal of Satellite Communications and Networking, 2023, 41(5): 441-459. DOI: 10.1002/sat.1477.
23. Reid TGR. Orbital diversity for global navigation satellite systems. Standford: Standford University, 2017.
24. Huang J, Liu Y, Liu X, et al. Optimal Design of LEO Constellation for Communication and Navigation Fusion Based on Genetic Algorithm. Lecture Notes in Electrical Engineering, 2021, Vol.773: 92-103. DOI: 10.1007/978-981-16-3142-9_9.
25. Yang Y, Yao Z, Mao Y, et al. Resilient satellite-based PNT system design and key technologies. Science China Earth Sciences, 2025,Vol.68(3): 669-682. DOI: 10.1007/s11430-024-1497-6.
26. Shtark T and Gurfil P. Position and velocity estimation with a low Earth orbit regional navigation satellite constellation. Proceedings of the Institution of Mechanical Engineers, Part G: Journal of Aerospace Engineering, 2022, 236(7): 1375-1387. DOI: 10.1177/09544100211031348.
27. Jiang C, Luo Z, Guan M, et al. Low orbit regional enhanced navigation constellation for BDS3 design based on Bayesian optimization algorithm. Geodesy and Geodynamics, 2024. DOI: 10.1016/j.geog.2023.12.003.
28. Xu S, Yang Q, Du X, et al. Multi-GNSS Precise Point Positioning enhanced by the real navigation signals from CENTISPACETM LEO mission. Advances in Space Research, 2024, 73(8): 4175-4186. DOI: 10.1016/j.asr.2024.01.017.
29. Qin JW, Mengda L, Shen N. Analysis of the Interception Ability of Electronic Reconnaissance Satellite on Ground-based Radars, 2021 6th International Conference on Intelligent Computing and Signal Processing (ICSP), Xi'an, China, 2021, pp. 698-701. DOI: 10.1109/ICSP51882.2021.9408736.
30. Huang H, An H, Wu B, et al. A non-nested collaborative optimization method for multidisciplinary design optimization and its application in satellite designs. PROCEEDINGS OF THE INSTITUTION OF MECHANICAL ENGINEERS PART G-JOURNAL OF AEROSPACE ENGINEERING, 2016, 230(12): 2292-2305.
31. Lukáš D, Kot T. Hierarchical Real-Time Optimal Planning of Collision-Free Trajectories of Collaborative Robots. Journal of Intelligent & Robotic Systems, 2023, 107(4). DOI: 10.1007/s10846-023-01848-9.
32. Lee Y, Choi J P. Connectivity analysis of mega-constellation satellite networks with optical

intersatellite links. IEEE Transactions on Aerospace and Electronic Systems, 2021, 57(6): 4213-4226. DOI: 10.1109/TAES.2021.3090914.
33. Zhang YL, Li Z, Shi C, et al. GDOP and Positioning Performance Analysis Based on Starlink, LEO Constellation. 13th China Satellite Navigation Annual Conference 2022-05-25.
34. Omid Y, Z. Bakhsh M, Kayhan F, et al. Space MIMO: Direct Unmodified Handheld to Multi-Satellite Communication, GLOBECOM 2023 - 2023 IEEE Global Communications Conference, Kuala Lumpur, Malaysia, 2023, pp. 1447-1452. DOI: 10.1109/GLOBECOM54140.2023.10437580.
35. Spangelo SC, Cutler JW, Klesh AT, et al. Models and Tools to Evaluate Space Communication Network Capacity. IEEE Transactions on Aerospace and Electronic Systems, vol. 48, no. 3, pp. 2387-2404, JULY 2012. DOI: 10.1109/TAES.2012.6237598.
36. Bakhsh ZM, Omid Y, Chen G, et al. Multi-Satellite MIMO Systems for Direct Satellite-to-Device Communications: A Survey. IEEE Communications Surveys & Tutorials, 2025. DOI: 10.1109/COMST.2024.3449430.
37. https://www.ucsusa.org/resources/satellite-database.
38. https://www.statista.com/statistics/897719/number-of-active-satellites-by-year/
39. https://newspaceeconomy.ca/2024/01/15/report-2/.
40. https://orbit.ing-now.com/.
41. Venkatesan A, Lowenthal J, Prem P, et al.. The impact of satellite constellations on space as an ancestral global commons . Nature Astronomy, 4, 1043–1048 (2020). DOI: 10.1038/s41550-020-01238-3.
42. McDowell JC. The low earth orbit satellite population and impacts of the SpaceX Starlink constellation. The Astrophysical Journal Letters, 2020, 892(2): L36. DOI: 10.3847/2041-8213/ab8016.
43. U.S. Government Accountability Office. Large Constellations of Satellites: Mitigating Environmental and Other Effects, GAO-22-105166, Sep 29, 2022.
44. Bassa CG, Hainaut OR and Galadí-Enríquez D. Analytical simulations of the effect of satellite constellations on optical and near-infrared observations. EDP Sciences, 2021. DOI: 10.1051/0004-6361/202142101.
45. Del Portillo I, Cameron BG, Crawley EF. A technical comparison of three low earth orbit satellite constellation systems to provide global broadband. Acta Astronautica, 2019, Vol.159: 123-135. DOI: 10.1016/j.actaastro.2019.03.040.
46. Rozenvasser D, Shulakova K. Estimation of the Starlink Global Satellite System Capacity. Proceedings of the International Conference on Applied Innovations in IT,2023,Vol.11(1): 55-59. DOI: 10.25673/101912.
47. Saleh JH, Torres Padilla JP. Beyond cost models: Communications satellite revenue models. Integrating cost considerations into a value-centric mindset. International Journal of Satellite Communications and Networking, 2007, 25(1): 69-92. DOI: 10.1002/sat.863.
48. Mahr E, Tu A, Gupta A. Development of the small satellite cost model 2014 (sscm14), in 2016 IEEE Aerospace Conference, pp. 1–13, IEEE, 2016. DOI: 10.1109/AERO.2016.7500515.
49. Radtke J, Kebschull C, Stoll E. Interactions of the space debris environment with mega constellations—Using the example of the OneWeb constellation. Acta Astronautica, 2017, 131: 55-68. DOI: 10.1016/j.actaastro.2016.11.021.
50. Steindl RM. Developing Detectability, Identifiability, and Trackability Analyses for the Space Sustainability Rating. Massachusetts Institute of Technology, Department of Mechanical Engineering, February, 2021.

51. Horstmann A, Hesselbach S, et al. Enhancement of S/C Fragmentation and Environment Evolution Models. European Space Agency, 2020.
52. ESA's fragmentation database [EB/OL]. https://sdup.esoc.esa.int.
53. Pardini C, Anselmo L. Using the space debris flux to assess the criticality of the environment in low Earth orbit. Acta Astronautica, 198, 2022, P:756-760. DOI: 10.1016/j.actaastro.2022.05.045.
54. Gusmini D, D'Ambrosio A, Servadio S, et al. Effects of Orbit Raising and Deorbiting in Source-Sink Evolutionary Models. JOURNAL OF SPACECRAFT AND ROCKETS, 2024. DOI: 10.2514/1.A35849.
55. Lemmens S, Letizia F. Space Traffic Management Through Environment Capacity. In: Schrogl, KU. (eds) Handbook of Space Security. Springer, Cham. 2020. DOI: 10.1007/978-3-030-23210-8_109.
56. Maury T, Loubet P, Trisolini M, et al. Assessing the impact of space debris on orbital resource in life cycle assessment: A proposed method and case study. Science of The Total Environment, 667, 2019, P:780-791. DOI: 10.1016/j.scitotenv.2019.02.438.
57. Liu SY, Guo XY, Lai J, et al. Distributed Timekeeping in BeiDou Inter-satellite Link Network. IEEE Communications Letters, 2022, 26(12): 1. DOI: 10.1109/LCOMM.2022.3198986.
58. Levy C, Pinchas M, Pinhasi Y. Coherent Integration Loss Due to Nonstationary Phase Noise in High-Resolution Millimeter-Wave Radars. Remote Sensing, 2021, 13(9). DOI: 10.3390/rs13091755.
59. Li SY, Xiao XD, Zheng XP. Distributed Coherent Aperture Radar Based on Microwave Photonics. Journal of Radar, 2019, 8 (2): 178-188. DOI: 10.1109/JLT.2020.3030668.
60. Antoniou M, Cherniakov M, Ma H. Space-surface bistatic synthetic aperture radar with navigation satellite transmissions: a review. Chinese Science (Information Science), 2015, 58 (6): 5-24. DOI: 10.1007/s11432-015-5334-6.
61. Kozhaya S, Kanj H, Kassas ZM. Multi-Constellation Blind Beacon Estimation, Doppler Tracking, and Opportunistic Positioning with OneWeb, Starlink, Iridium NEXT, and Orbcomm LEO Satellites. 2023 IEEE/ION Position, Location and Navigation Symposium (PLANS), 2023.
62. Mechalikh C, Taktak H, Moussa F. PureEdgeSim: A Simulation Toolkit for Performance Evaluation of Cloud, Fog, and Pure Edge Computing Environments. 2019 International Conference on High Performance Computing & Simulation (HPCS),2019.
63. Brandenburg FJ, Cai MC. Shortest path and maximum flow problems in networks with additive losses and gains. Theoretical Computer Science, 2011, 412(4). 391-401. DOI: 10.1016/j.tcs.2010.11.019.
64. Maiolini Capez G, Cáceres MA, Armellin RP, et al. Characterization of Mega-Constellation Links for LEO Missions With Applications to EO and ISS Use Cases. IEEE Access, 2023,Vol.11: 25616-25628. DOI: 10.1109/ACCESS.2023.3254917.
